# The AraC-Type Transcriptional Regulator GliR (PA3027) Activates Genes of Glycerolipid Metabolism in *Pseudomonas aeruginosa*

**DOI:** 10.3390/ijms22105066

**Published:** 2021-05-11

**Authors:** Karolina Kotecka, Adam Kawalek, Kamil Kobylecki, Aneta Agnieszka Bartosik

**Affiliations:** Institute of Biochemistry and Biophysics, Polish Academy of Sciences, 02-106 Warsaw, Poland; ka.kotecka@ibb.waw.pl (K.K.); a.kawalek@ibb.waw.pl (A.K.); kkobylecki@ibb.waw.pl (K.K.)

**Keywords:** *Pseudomonas aeruginosa*, AraC family, transcriptional regulator, PA3027, regulon, glycerolipid metabolism

## Abstract

*Pseudomonas aeruginosa* encodes a large set of transcriptional regulators (TRs) that modulate and manage cellular metabolism to survive in variable environmental conditions including that of the human body. The AraC family regulators are an abundant group of TRs in bacteria, mostly acting as gene expression activators, controlling diverse cellular functions (e.g., carbon metabolism, stress response, and virulence). The PA3027 protein from *P. aeruginosa* has been classified in silico as a putative AraC-type TR. Transcriptional profiling of *P. aeruginosa* PAO1161 overexpressing *PA3027* revealed a spectacular increase in the mRNA levels of *PA3026*-*PA3024* (divergent to *PA3027*), *PA3464*, and *PA3342* genes encoding proteins potentially involved in glycerolipid metabolism. Concomitantly, chromatin immunoprecipitation-sequencing (ChIP-seq) analysis revealed that at least 22 regions are bound by PA3027 in the PAO1161 genome. These encompass promoter regions of *PA3026*, *PA3464*, and *PA3342*, showing the major increase in expression in response to PA3027 excess. In Vitro DNA binding assay confirmed interactions of PA3027 with these regions. Furthermore, promoter-reporter assays in a heterologous host showed the PA3027-dependent activation of the promoter of the *PA3026*-*PA3024* operon. Two motifs representing the preferred binding sites for PA3027, one localized upstream and one overlapping with the −35 promoter sequence, were identified in *PA3026*p and our data indicate that both motifs are required for full activation of this promoter by PA3027. Overall, the presented data show that PA3027 acts as a transcriptional regulator in *P. aeruginosa*, activating genes likely engaged in glycerolipid metabolism. The GliR name, from a glycerolipid metabolism regulator, is proposed for PA3027 of *P. aeruginosa*.

## 1. Introduction

The abundance of transcriptional regulators in bacteria allows for the optimization of gene expression and response to stimuli. Nineteen prokaryotic transcriptional regulator families have been evaluated thus far [1]. The most abundant groups in bacterial genomes include the LysR, TetR/AcrR, AraC/XylS, and Lrp families. The ample repertoire of transcriptional regulators (TRs) is a characteristic feature of free-living microorganisms and opportunistic pathogens, in contrast to intracellular organisms.

*Pseudomonas aeruginosa* is a ubiquitous bacterium, also known for being a facultative human pathogen, especially dangerous for immunocompromised patients, mainly due to its genetic plasticity and intrinsic antibiotic resistance mechanisms [2]. The *P. aeruginosa* genome encodes complex regulatory systems, which include more than 500 known and potential transcriptional regulators or two-component system proteins [3], altogether constituting almost 10% of all its genes. The regulatory network of this bacterium allows it to modulate and manage cellular metabolism to survive in variable environmental conditions including the human body, causing infections of lungs, wounds, blood, and urinary tracts [4]. Recent studies performed by Huang and co-workers [5] showed how 20 key virulence-related transcriptional regulators from different TR families work, crosstalk, and affect transcription of target genes in *P. aeruginosa*. It highlighted how complicated the regulatory network is and how many factors influence the regulation of gene expression at any given moment.

The AraC family regulators are an abundant group of TRs in bacteria, mostly acting as gene expression activators [6]. The representatives of this family possess a highly conserved C-terminal domain containing two helix-turn-helix (HTH) motifs responsible for DNA-binding, and a variable N-terminal domain, called the ligand-binding domain (LBD) or response domain, involved in interactions with cognate ligands as well as participating in protein oligomerization.

The well characterized archetype of this family is the AraC activator of the *araBAD* operon, which is involved in the metabolism of L-arabinose in *Escherichia coli* [7]. Its action strongly depends on arabinose binding [8]. There are three AraC binding sites in the *araBAD* promoter: I_1_ and I_2_, located next to RNA polymerase binding sites, and the distant upstream site named O_2_. In the absence of arabinose, AraC binds to I_1_ and O_2_ and forms a dimer, which causes DNA looping. This conformation prevents RNA polymerase from binding to the *araBAD* promoter, so the AraC acts as a repressor. When arabinose is present, AraC binds the ligand and changes conformation. As a result, the DNA loop breaks and AraC monomers bind to I_1_ and I_2_ sites and help to recruit RNA polymerase to the promoter to initiate transcription [9].

The mode of action of AraC-type regulators was shown to be controlled by different mechanisms (e.g., the oligomeric state and/or conformation can be changed after ligand binding but also upon interaction with a partner protein) [6]. In the absence of ligands, they can act as repressors, while after ligand binding and conformation changes, they can act as the activators [6]. The ligands of AraC-type regulators are usually small organic compounds like sugars (e.g., arabinose for *E. coli* AraC [7], lactose for *Clostridium perfringens* BgaR) [10], amino acids, and their derivatives (e.g., sarcosine for SouR (PA4184) from *P. aeruginosa*) [11], or other small molecules (e.g., urea for UreR in *Providencia stuartii*) [12]. The AraC-type regulators control diverse cellular functions including carbon metabolism (e.g., GapR activates the transcription of the *gap* gene encoding glyceraldehyde-3-phosphate dehydrogenase in S*treptomyces aureofaciens*) [13]; type III secretion systems (e.g., VirF in *Shigella* spp., HilC/HilD in *Salmonella* spp., SPI-1, or ExsA in *P. aeruginosa*) [14]; stress response (e.g., Rob, SoxS, PilA, or OpiA in *Erwinia* *amylovora*) [15]; quorum sensing and virulence (e.g., QsvR in *Vibrio parahaemolyticus*) [16].

*P. aeruginosa* PAO1 encodes 16 known and 41 potential TRs from the AraC/XylS family (based on NCBI and pseudomonas.com databases), which makes this group one of the largest among TR families in *P. aeruginosa*. Most of the AraC family TRs thus far characterized in this bacterium are described as involved in the regulation of metabolism: OruR (PA0831) is an ornithine degradation activator [17], ArgR (PA0893) controls arginine biosynthesis and aerobic catabolism [18], AntR (PA2511) is an activator of anthranilate degradation [19], MmsR (PA3571) is a positive regulator of amino acid biosynthesis [20], SouR (PA4184) is essential for growth on sarcosine [11], PchR (PA4227) regulates pyochelin biosynthesis [21], GbdR (PA5380) controls choline metabolism [22], CdhR (PA5389) regulates carnitine metabolism [23], and PruR (PA0780) is a proline utilization regulator important for virulence [24]. Some of the TRs from the AraC/XylS family play different roles in *P*. *aeruginosa* virulence, for example, ChpD (PA0416) was required for spreading and colonizing the liver [25]; ExsA (PA1713) played a role in colonization of the corneal epithelium [26]; VqsM (PA2227), a positive regulator of quorum sensing (QS), was also shown to be involved in modulation of antibiotic resistance and biofilm formation [27,28]; CdpR (PA2588), a positive regulator of pyocyanin and biofilm production [29]; SphR (PA5324), an activator of *sphA* gene important for survival in the murine lung as well as for resistance to the antimicrobial effect of the pulmonary surfactant sphingosine [30], or CmrA (PA2047) involved in activation of *mexEF*–*oprN* and increased resistance of *P. aeruginosa* to the pump substrates such as chloramphenicol, fluoroquinolones, or trimethoprim [31].

The PA3027 protein from *P. aeruginosa* has been classified in silico as a putative AraC/XylS-type transcriptional regulator. The *PA3027* gene was previously identified as downregulated in *P. aeruginosa parA* and *parB* mutants, which showed disturbed chromosome segregation [32,33,34]. The genes with altered expression in *par* mutants represented different functional categories, however, a significant number of genes encoding transcriptional regulators, often of unknown functions, was noted [32,35]. This study aimed to decipher the function of one of them, the AraC-type transcriptional regulator PA3027.

## 2. Results

### 2.1. Overview of the PA3027 from P. aeruginosa PAO1161

**Figure 1:**
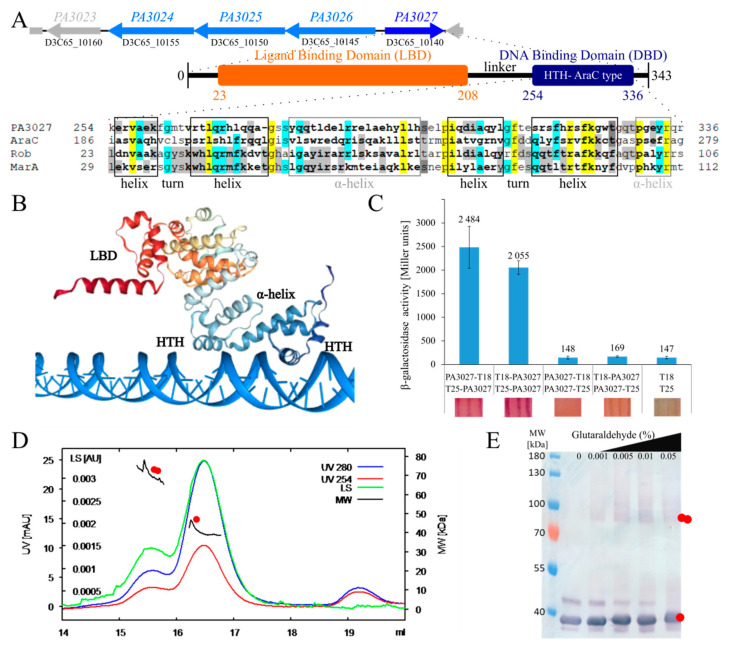
Properties of PA3027 protein from *P. aeruginosa*. (**A**) Genomic context of the *PA3027* gene in the *P. aeruginosa* genome and domain structure of the PA3027 protein. The gene names from PAO1 and PAO1161 strains are presented. Alignment represents comparison of PA3027 HTH domain with corresponding regions of *E*. *coli* AraC (GenBank: CAA23508.1*)*, Rob (GenBank: CAD6017604.1), and MarA (GenBank: AAK21293.1). Sequences were aligned using Clustal Omega [36]. Identical residues in all proteins were marked with yellow, in three sequences with blue and in two with grey. The secondary structure elements are marked with boxes based on MarA secondary structure [37]. (**B**) Structure of PA3027 monomer bound with DNA predicted using COACH and HDOCK [38,39,40]. LBD—ligand binding domain; HTH— helix-turn-helix. (**C**) Bacterial two-hybrid (BACTH) analysis of PA3027 self-interactions. *E. coli* BTH101 *cya*^−^ was transformed with the pairs of vectors allowing expression of the indicated fusion proteins. Interactions between proteins were assayed by analysis of the β-galactosidase activity in cell extracts and analysis of colony color upon growth on McConkey medium with 1% maltose. Data indicate mean β-galactosidase activity from at least three replicates ±SD. (**D**) Size exclusion chromatography (SEC) with multi-angle static light scattering (MALS) analysis for His_6_–PA3027. Left axis—UV absorption and light scattering (LS), right axis—molecular weight of protein (MW). (**E**) Oligomerization state of purified His_6_-PA3027 assayed by crosslinking with increasing concentration of glutaraldehyde. Samples were used in Western blot analysis with anti-His antibodies. For (**D**,**E**), one red dot indicates a monomer and two dots indicate a dimer.

The *PA3027* gene in *P. aeruginosa*, transcribed divergently to the *PA3026–PA3022* cluster, encodes a protein classified in silico as a putative AraC-type TR with two predicted domains: the N-terminal ligand-binding domain (LBD) and the C-terminal DNA binding domain with a HTH motif (Figure 1A). The region of PA3027 with the highest similarity to well-described members of the AraC family: AraC, Rob, and MarA from *E. coli*, was identified in the C-terminal part of the protein encompassing the putative HTH containing region (Figure 1A).

The secondary structure prediction showed two potential HTH DNA binding motifs in the C-terminus of PA3027 (Figure 1A) [37]. The first HTH motif was predicted to be located at position 256–284, while the second one was predicted to be formed by residues 298 to 335. Similarly, PA3027 protein structure prediction using COACH and HDOCK [38,39,40] also suggested the existence of two possible HTH motifs involved in contact with DNA (Figure 1B). These data indicate the sequence similarity between PA3027 and other AraC-type TRs.

To analyze the oligomeric state of PA3027, various methods were applied. The bacterial two-hybrid analysis revealed that PA3027 can self-assemble in vivo, but only in the case when the-C-terminal part of the protein is free in at least one of the tested fusions (variants T18–PA3027/T25–PA3027 and PA3027–T18/T25–PA3027) (Figure 1C). Concomitantly, size-exclusion chromatography combined with multi-angle light scattering (SEC-MALS) analysis with purified His_6_-PA3027 demonstrated that this protein existed preferentially as a monomer, but it could also form dimers in solution under the tested conditions (Figure 1D). Similarly, glutaraldehyde crosslinking of purified His_6_–PA3027 followed by Western blot analysis also showed the presence of protein dimers (Figure 1E). These data indicate that like other AraC-type regulators, PA3027 may exist as a monomer or dimer.

### 2.2. Effect of Increased PA3027 Level on Gene Expression

**Figure 2:**
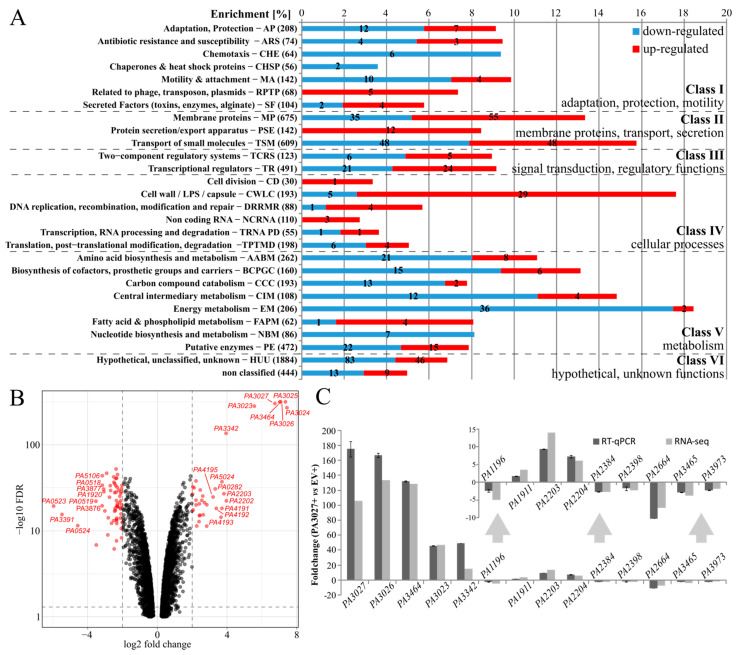
Effect of increased PA3027 level on gene expression in *P. aeruginosa* PAO1161 cells. (**A**) Enrichment of PseudoCAP functional categories [41] for 539 genes (306 downregulated; 233 upregulated) showing changes in mRNA level in response to PA3027 abundance (fold change ≤ −2 or ≥ 2, FDR adjusted *p*-value ≤ 0.01). The numbers in brackets show the number of all genes in the PAO1 genome in the indicated PseudoCAP category. One gene could be classified into more than one category or class. Numbers in red or blue bars represent the number of up- or downregulated genes, respectively, in each category. The PseudoCAP categories were grouped into six more general classes. Genes annotated only in PAO1161 strain but not in PAO1 are described as non classified. (**B**) Volcano plot visualization of the results of differential expression analysis between transcriptomes of PA3027 overproducing cells and control cells. Each dot represents one gene and genes with the most significant changes are colored in red. For clarity genes with *p*-value < 0.1 are not shown. (**C**) Validation of RNA-seq results by RT-qPCR analysis. The same RNA used for RNA-seq analysis was used for cDNA synthesis and RT-qPCR analysis. Data represent mean fold change for three samples of PA3027 overproducing cells relative to the mean of the control samples ± SD.

**Figure A1:**
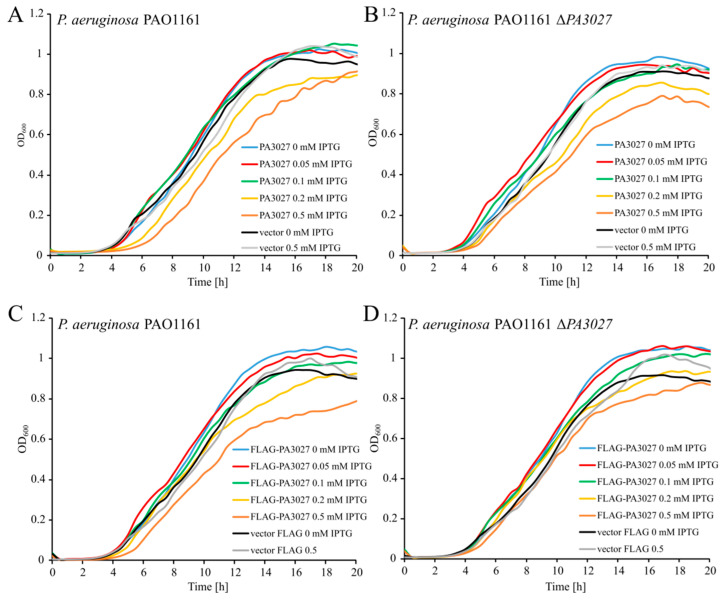
Impact of PA3027 excess on the growth of *P. aeruginosa* PAO1161. PAO1161 or Δ*PA3027* mutant strains carrying empty vector pAMB9.37 *tac*p or pKKB1.11-*tac*p-*PA3027* (**A**,**B**) or empty vector *tac*p–*flag* or pKKB1.12 *tac*p–*flag*–*PA3027* (**C**,**D**) were grown in L-broth under selection with the indicated concentration of inducer IPTG (0.05–0.5 mM). The red line indicates the growth in the presence of 0.05 mM IPTG, conditions selected for RNA-seq analysis. Data represent mean OD_600_ from three independent replicates. Standard deviations are not shown for clarity.

AraC-type TRs may act as gene expression activators but also as repressors. To identify genes regulated by PA3027 in *P*. *aeruginosa*, RNA-sequencing was performed under conditions of slight PA3027 overproduction, not affecting the growth of the cells (Appendix A Figure A1A). The rationale behind the analysis of cells with PA3027 excess rather than the Δ*PA3027* mutant was based on (1) relatively low expression of *PA3027* under standard growth conditions (LB or M9 medium, data not shown); (2) using protein excess could possibly mimic the induced, activated state of the protein; and (3) the effector for this regulator is unknown. Comparison of the transcriptomes of PAO1161 cells carrying pKKB1.11 (*tac*p–*PA3027*, hereafter called PA3027+) and PAO1161 cells with pAMB9.37 (*tac*p, empty vector control, hereafter called EV+), grown under selection in L broth with 0.05 mM IPTG, demonstrated 539 loci with altered expression (fold change (FC) ≤ −2 or ≥ 2, FDR adjusted *p*-value ≤ 0.01) (Figure 2A; Table S1). A total of 306 loci were downregulated, while 233 showed increased expression. The genes with altered mRNA levels were assigned to PseudoCAP functional categories [41] and grouped arbitrarily into six more general classes, as described previously [32,42]. The majority of identified genes belonged to classes II, IV, and V, and the highest enrichment was observed for the following categories: energy metabolism (18%, mostly downregulated genes), cell wall/LPS/capsule (18%, mostly upregulated genes), and transport of small molecules (16%) (Figure 2A). These data indicate that PA3027 excess in *P. aeruginosa* influenced the expression of genes from different functional categories.

The volcano plot visualization of the results of differential expression analysis highlighted genes with the most significant changes in mRNA level (Figure 2B). A spectacular increase in expression (>40) in response to PA3027 excess was observed for the *PA3026*–*PA3023* gene cluster, transcribed divergently to the *PA3027* (Figure 1A) as well as *PA3464* gene (Figure 2B, Table 1). These genes encode proteins, with predicted functions in glycerolipid metabolism: an oxidoreductase acting on CH–OH group of donors (PA3026), a glycerol-3-phosphate dehydrogenase (PA3025), a carbohydrate kinase (PA3024), a diacylglycerol/lipid kinase (PA3023), and a phospholipase C (PA3464). Concomitantly, a high decrease in expression in PA3027+ cells relative to EV+ cells was observed for the genes encoding cytochrome c (*PA0523*) as well as the regulatory protein NosR; both involved in the regulation of expression of the nitrous oxide reductase gene *nosZ* [43] (Table 1). Essentially, RT-qPCR (reverse transcription followed by quantitative PCR) analysis, using the material used for RNA-seq analysis validated the observed changes in mRNA level of chosen genes, confirming the influence of PA3027 on their expression (Figure 2C).

### 2.3. Identification of PA3027 Binding Sites in P. aeruginosa

**Figure 3:**
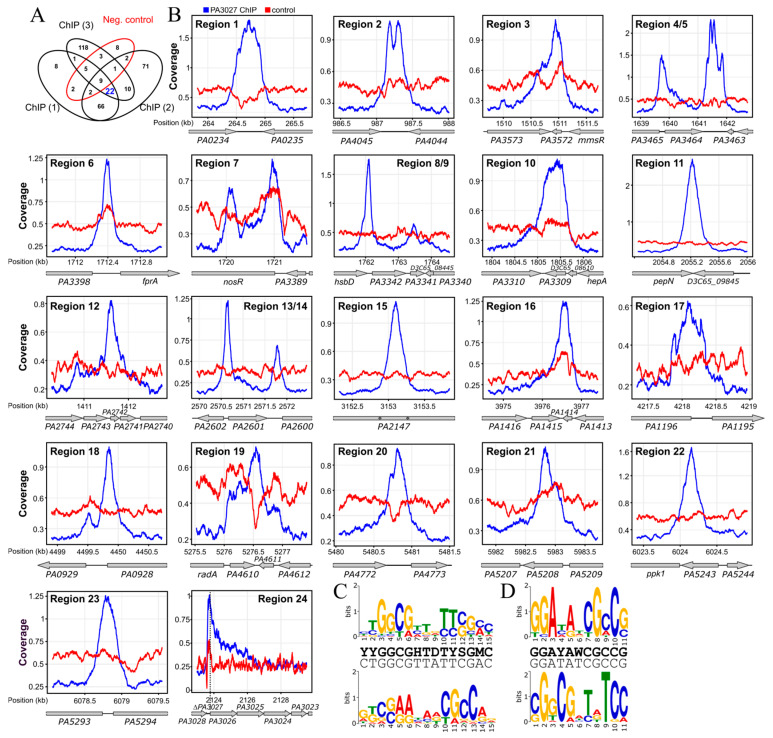
PA3027 binding sites in *P. aeruginosa* genome. (**A**) Venn diagram for ChIP-seq peaks obtained for samples of FLAG-PA3027 overproducing cells (F–PA3027+) and negative control (F–EV+). (**B**) ChIP-seq signal over regions encompassing PA3027 binding sites. The plots show coverage with reads for indicated positions in the PAO1161 genome (kb), normalized per genome coverage (RPGC), and averaged for ChIP replicates. Genes are presented as grey arrows, only names of PAO1 orthologs are shown for clarity. (**C**,**D**) The consensus sequence logos of predicted PA3027 binding sites, obtained by MEME software [44,45] using 200 bp around 24 PA3027 peak summits (**A**) as well as the same 24 PA3027 peak summit regions with an extended to 500 bp 24 region encompassing *PA3026* upstream sequences (**B**). The height of an individual letter represents the relative frequency of that nucleotide at that position. The consensus sequence (up line) and the most common nucleotide at each position (down line) are presented for each motif. The reverse complement presentation of sequence logos are shown below.

To identify the PA3027 binding sites in the PAO1161 genome and hence the direct targets of the regulator, a chromatin immunoprecipitation-sequencing (ChIP-seq) analysis was performed using PAO1161 Δ*PA3027* pKKB1.12 (*tac*p–*flag*–*PA3027*) strain expressing *flag*–*PA3027* under the control of *tac*p (hereafter called F–3027+). The addition of the FLAG tag to PA3027 did not alter the ability of the protein to retard bacterial growth in medium with a high (0.5 mM) concentration of IPTG (Figure A1), suggesting that the fusion protein is functional. The PAO1161 Δ*PA3027* (pABB28.1 *tac*p–*flag*) strain was used as an empty vector control (F–EV+). ChIP-seq was performed using cells grown under selection in L broth with 0.05 mM IPTG to OD_600_ of 0.5 and anti-FLAG antibodies. Sets of ChIP-seq peaks called separately for each of the three F–3027+ ChIP replicates using the FDR adjusted *p*-value cut-off of 0.05 and fold enrichment cut-off 2 (Table S2A–C) were compared and peaks in genome regions also showing enrichment in the F–EV+ ChIP samples were discarded (Table S2D; Figure 3A). Overall, this analysis pointed out 22 high confidence PA3027 binding sites in the *P. aeruginosa* PAO1161 genome (Figure 3B). Two FLAG-PA3027 bound regions, 4/5 and 13/14, showed ChIP-seq peaks with two clearly separated summits, hence these were considered as separate binding sites in subsequent analyses (Table 2). Interestingly, these sites mapped to the promoter region as well as the terminator region of the flanked gene (Figure 3B).

The identified PA3027 binding sites were compared with RNA-seq data to define genes possibly directly regulated by PA3027 binding in their vicinity (Table 2). Six PA3027 binding sites mapped to the promoter regions, five in potential terminator regions and 12 were identified in the gene bodies (Table 2). The PA3027 binding site with the highest fold enrichment encompasses the downstream region/terminator as well as the promoter region of the *PA3464* gene, which also showed major upregulation in response to PA3027 excess (Figure 3B, region 4/5). PA3027 binding sites were also detected in regions preceding *PA3026* and *PA3342* genes, similarly, demonstrating increased expression in RNA-seq analysis (Figure 3B, region 24 and 8/9). Concomitantly, PA3027 binding regions were also identified within gene bodies, and some of these seemed to exert an effect on neighboring gene expression, as shown in the RNA-seq analysis for *PA3391* (*nosR*), *PA3572*, *PA3309*, *PA1414*, *PA1196*, *PA4610*, *PA5208*, all exhibiting a decreased expression in response to PA3027 excess (Table 2; Figure 3B).

**Figure 4:**
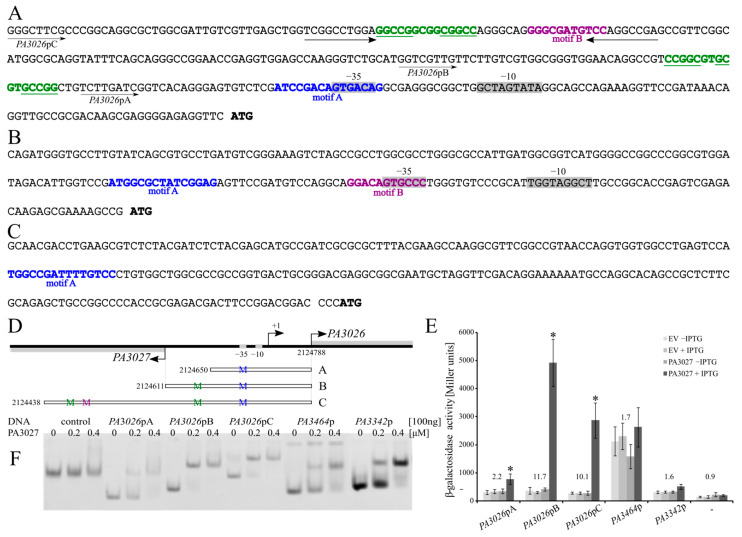
PA3027 interaction with DNA assayed in vivo and in vitro. (**A**–**C**) Putative PA3027 binding motifs in *PA3026* (**A**), *PA3464* (**B**), and *PA3342* (**C**) promoter fragments, used in β-galactosidase activity assays and electrophoretic mobility shift assay (EMSA) analysis. Blue—motif A, violet—motif B, green—pseudoplindrome, grey—“−10” and “−35” promoter regions. (**D**) The scheme of variants of the *PA3026–PA3024* promoter used in the analysis and cloned to pCM132 upstream of a promoter-less *lacZ* reporter gene. (**E**) Influence of PA3027 on the activity of *PA3026*p, *PA3464*p, and *PA3342*p. β-galactosidase activity in extracts from *E. coli* DH5α Δ*lac* cells bearing pCM132 derivatives containing the indicated promoters upstream of *lacZ* as well as pKKB1.11 (*tac*p-PA3027), allowing IPTG inducible PA3027 production or empty vector pAMB9.37 (EV). Strains were cultured in medium with or without 0.1 mM IPTG. Data indicate mean β-galactosidase activity ±SD from five cultures. * *p*-value < 0.05 in student’s two tailed *t*-test. (**F**) EMSA using His_6_–PA3027 and PCR amplified DNA of the indicated promoter regions. The 100 ng Cy5 tagged DNA was incubated with an increasing amount of His_6_-PA3027. Samples were separated using 10% acrylamide gel and Cy5 fluorescence was visualized. The 331 bp pCM132 fragment was used as a control to rule out non-specific DNA binding.

To identify recurrent DNA sequences in ChIP-seq peaks and define the PA3027 consensus-binding site, the MEME tool [45] was applied on 24 sequences corresponding to the PA3027 ChIP-seq peak summits ±100 bp sequences as well as the same 24 PA3027 peak summit regions with an extended to 500 bp region 24 encompassing *PA3026* upstream sequences. Two sequences called here motif A (15 bp) and motif B (11 bp) were identified with proposed consensus sequences YYGGCGHTDTYSGMC and GGAYAWCGCCG, respectively (Figure 3C,D). Interestingly, in the reverse complement orientation, a part of motif B resembles a part of motif A. The localization of identified motifs within promoter regions of activated genes (*PA3026*, *PA3364*, and *PA3342*) showed their presence upstream, or even overlapping the predicted –35 promoter region, a position preferred for binding by transcriptional activators (e.g., AraC, MarA) [9,46] (Figure 4A–C). The localization of identified motifs in 24 regions detected as bound by PA3027 is presented in Appendix A Table A1. Additionally, in the promoter region of the *PA3026* gene, next to motifs A and B, partial palindromes CCGGCGTGCGTGCCGG and GGCCGGCGGCGGCC as well as inverted repeat TCGGCCTGGA-N29-TCCAGGCCGA had been noticed (Figure 4A). Partially, they resemble the identified PA3027 binding motifs and potentially might be involved in DNA recognition and binding by PA3027.

### 2.4. Regulatory Properties of PA3027

To evaluate the regulatory properties of PA3027, the *PA3026* promoter region was selected to which PA3027 showed strong binding, and a spectacular increase in *PA3026* expression was observed in response to PA3027 excess. Three variants of the *PA3026* promoter region were cloned in the probing vector pCM132 [47], carrying a promoter-less *lacZ* (Figure 4D). All tested variants were able to act as promoters for the *lacZ* reporter gene, as manifested by higher β-galactosidase activity in cells carrying *PA3026*p-*lacZ* fusions in comparison to the promoter-less *lacZ* control (Figure 4E). No change of *lacZ* expression driven from *PA3026*p variants was observed in the absence or the presence of IPTG, when no PA3027 was delivered (EV), or in the cultures without PA3027 induction (–IPTG). Essentially, an addition of IPTG promoting *PA3027* expression led to a major increase in the *PA3026* promoter activity (Figure 4E). The lowest induction, approximately 2-fold, was observed for the shortest *PA3026*pA fragment. For fragment *PA3026*pB, the highest β-galactosidase activity and the highest induction (almost 12-fold) was observed, in comparison to promoter activity without PA3027 induction. A slightly lower, (10-fold) increase was observed for the *PA3026*pC-*lacZ* fusion. Importantly, the identified PA3027 binding motifs (Figure 3C,D), were present in all tested fragments of *PA3026*p, however, in different numbers. Only one PA3027 binding motif A, encompassing the −35 region of *PA3026*p, was present in the shortest tested fragment A. The pseudopalindrome located 38 bp upstream of −35 box of *PA3026*p was additionally present in fragment B, and an additional distal putative PA3027 binding motif, resembling motif B, located 162 upstream to the −35 promoter region was present in *PA3026*p*C* (Figure 4A).

Essentially, all the DNA fragments used in the regulatory experiments were bound by His_6_-PA3027 in the electrophoretic mobility shift assay (EMSA) (Figure 4F), whereas no DNA shift of the control fragment (part of pCM132) was observed. The binding to the shortest version of the *PA3026*p promoter (fragment A) was somewhat weaker than for other tested *PA3026*p variants (Figure 4F). These data demonstrate the ability of PA3027 to activate *PA3026*p in a heterologous host and highlights the requirement of sequences upstream of the −35 promoter region for full activation of this promoter by PA3027.

Similar analysis with cells expressing *PA3464*p-*lacZ* or *PA3342*p-*lacZ* only showed a minor increase in expression from the tested promoters in the presence of PA3027 (Figure 4E), whereas DNA fragments with these sequences were clearly bound in the EMSA experiments. Thus, it is not excluded that additional factors or upstream/downstream sequences are required for the control of the expression of *PA3464* and *PA3342* by PA3027 in *P. aeruginosa*. Overall presented data confirm PA3027 interactions with promoter regions of *PA3026*, *PA3464*, and *PA3342* and highlight its role as a DNA-binding protein involved in transcription control.

### 2.5. Toward the Biological Function of PA3027-PA3026-PA3023 Gene Cluster

**Figure 5:**
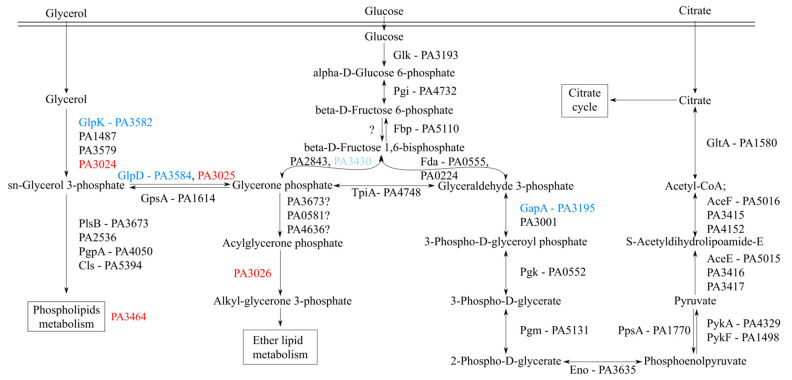
Schematic representation of the part of central carbon metabolism in *P. aeruginosa*. The pathways were drawn based on the Pseudomonas database [48] and the literature [49,50,51]. Red and blue indicate increased or decreased gene expression in response to PA3027 overproduction, respectively.

In silico analyses and database mining suggested that the products of the *PA3026*–*PA3024* operon are potentially involved in the transformation of glycerol to alkylglycerone phosphate (Figure 5). PA3024 is probably involved in the conversion of glycerol to sn-glyceryl-3-phosphate. In *P. aeruginosa*, this reaction could also be catalyzed by GlpK, PA1487, and PA3579. The second protein, PA3025, is a homolog of GlpD, and possibly catalyzes the conversion of sn-glyceryl-3-phosphate to glycerone phosphate. The enzymes catalyzing the next reaction, converting glycerone phosphate to acylglycerone phosphate are unknown in *P. aeruginosa*, but the potential candidates could be genes encoding GNPAT–glyceronephosphate O-acyltransferases such as PA3673 (PlsB), PA0581, or PA4636. The last protein encoded in the *PA3026*–*PA3024* operon could possibly facilitate conversion of acylglycerone phosphate to alkyl-glycerone phosphate. This analysis suggests that there might be a functional redundancy of enzymes encoded by *PA3026*–*PA3024* and other proteins in *P. aeruginosa*.

**Figure A2:**
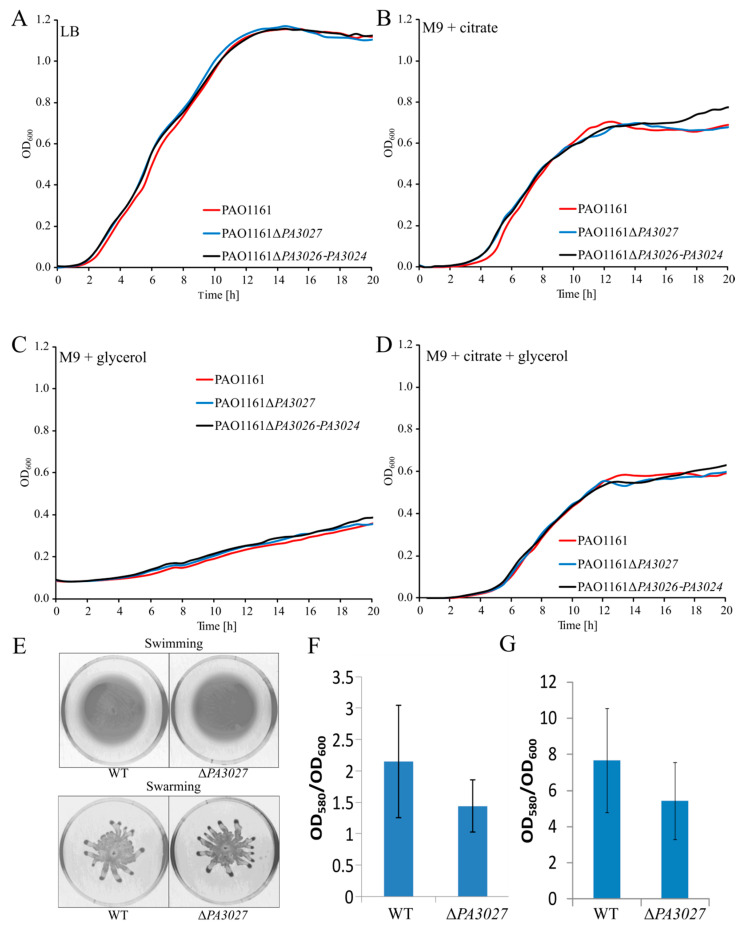
Phenotypic characterization of *P. aeruginosa* PAO1161 cells lacking *PA3027* or *PA3026*–*PA3024* operon. (**A**–**D**) Growth of PAO1161 *leu*^+^ and Δ*PA3027* mutant on (**A**) L broth, (**B**) minimal medium with citrate, (**C**) minimal medium with glycerol, or (**D**) minimal medium with citrate and glycerol. Data represent OD_600_ mean from three independent replicates. (**E**) Growth of PAO1161 and Δ*PA3027* mutant on swimming and swarming medium. (**F**,**G**) Biofilm formation of PAO1161 *leu^−^* and Δ*PA3027* mutant on (**F**) L broth or (**G**) minimal medium with citrate. Data represent mean from five independent replicates.

In an attempt to assign a biological function to PA3027, the chromosomal mutants of PAO1161 in *PA3027* and *PA3026*-*PA3024* genes (the whole operon deleted) were obtained using the allele exchange method. The ability to generate the Δ*PA3027* and Δ*PA3026*–*PA3024* strains indicates that the genes are not essential for the growth of *P. aeruginosa*. Lack of *PA3027* or *PA3026*-*PA3024* did not affect the growth of the cells on rich L broth medium, or minimal medium M9 supplemented with citrate and/or glycerol as the carbon source (Figure A2A–D), or in osmotic stress medium, a minimal medium with 0.5 M NaCl or 0.7 M sucrose (data not shown). Moreover, a BIOLOG microarray phenotype screening with plate no. 1, 2, 3, 4, 9, 10, 15, 16, and 18 did not show any effect of the gene deletion(s) on growth (data not shown) [52]. Additionally, no changes in bacterial motility or biofilm formation were observed between Δ*PA3027* mutant and the WT strain (Figure A2E–G). These data indicate that under the conditions tested, the analyzed genes do not play a major role in cell fitness or that there is a backup pathway compensating the lack of these genes.

**Figure A3:**
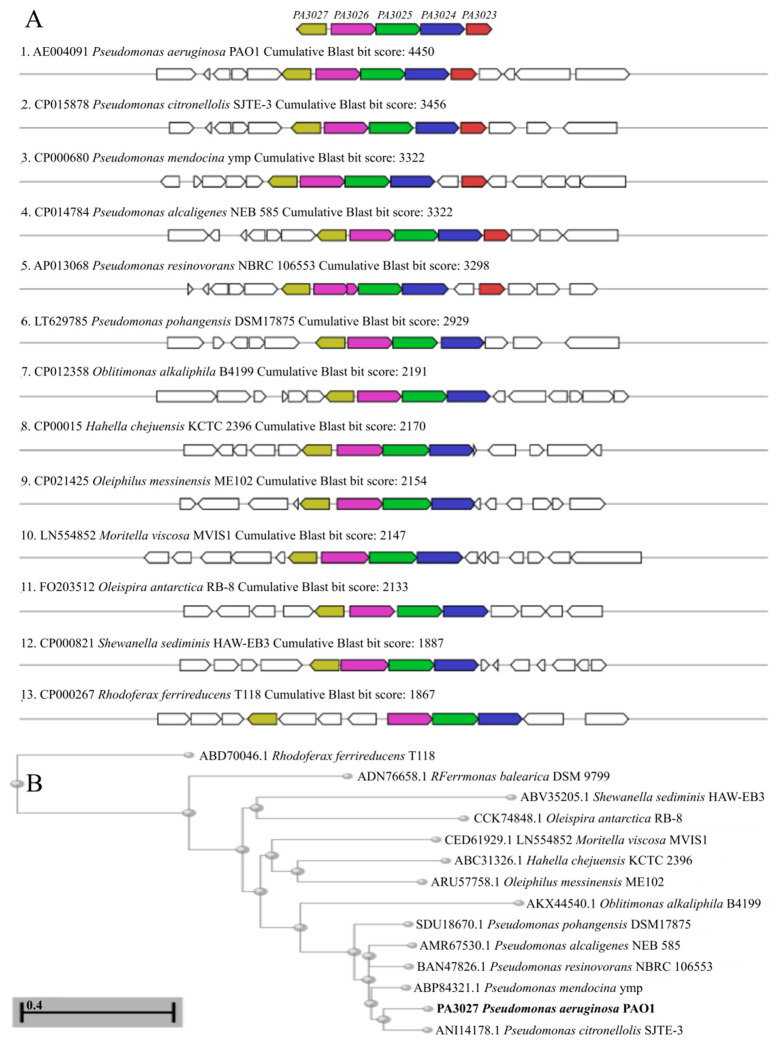
The occurrence of *PA3023*–*PA3027* gene cluster in bacteria. (**A**) Clustered genes encoding orthologs of *PA3023*–*PA3027* identified in 1748 representative and reference bacterial genomes included in the RefSeq database (release 91) [98] using MultigeneBlast [97]. (**B**) Phylogenetic tree of PA3027 and its identified orthologues, constructed using CoBaltDB [99].

A search of genes encoding orthologs of proteins encoded by the *PA3027-PA3023* gene cluster in 1748 representative and reference bacterial genomes included in the RefSeq database (release 91) using MultiGeneBlast revealed only four genomes encoding clustered orthologs of all analyzed proteins (Figure A3A; Table S3). Among these, two genomes possessed the gene encoding a PA3023 orthologue separated from the rest by an additional gene: a protein with a pirin domain for *Pseudomonas mendocina* and a potential oxidoreductase for *Pseudomonas resinovorans*. Another eight strains demonstrated a similar arrangement of the PA3027 and PA3026–PA3024 orthologue encoding genes. The strains belong mostly to the genus *Pseudomonas* as well as other gamma-proteobacteria (e.g., *Oblitimonas alkalophila* or *Shewanella sediminis*) and one representative of the beta-proteobacteria (*Rhodoferax ferrireducens*). In *R. ferrireducens*, the PA3027 encoding orthologue is separated from the operon by genes encoding a glycerate 2-kinase, an unknown protein and a lipolytic enzyme. Comparison of the orthologs of the PA3027 transcriptional regulator from identified organisms showed the highest similarity among proteins encoded in *Pseudomonas* sp. genomes (Figure A3B). The most similar gene to PA3027 is the TR from *P. citronellolis* P3B5, which was isolated from ready-to-eat basil (*Ocimum basilicum*) phyllosphere material [53]. These data indicate that the presence of genes encoding PA3027–PA3024-like proteins is not unique to PAO1/PAO1161 or *Pseudomonas* and occurs in other bacteria, however, only in a very limited number of so far sequenced strains. Interestingly, among the identified strains outside the genus *Pseudomonas*, no homologs of *glpD* or *glpK* were identified, thus *PA3025* and *PA3024* may encode the only glycerol-3-phosphate dehydrogenase and carbohydrate kinase, respectively. These data suggest the involvement of PA3027 and PA3026–PA3024 proteins in glycerolipid metabolism, however, further studies are needed to assign a precise biological role to these proteins.

## 3. Discussion

The aim of this study was a multilayered analysis of PA3027 from *P. aeruginosa,* a previously uncharacterized AraC-type transcriptional regulator [54]. The sequence analysis showed that PA3027 possesses a tandem HTH motif in the C-terminal part of the protein (Figure 1A), indicating the existence of two surfaces involved in contact with DNA, which is typical for AraC-type regulators [6]. Additionally, SEC-MALS and glutaraldehyde crosslinking experiments showed that despite PA3027 being preferentially a monomer under the conditions tested, the protein may also form dimers (Figure 1D,E). Bacterial two-hybrid analysis revealed the lack of self-interactions for the PA3027-T18/PA3027-T25 pair, suggesting that the free C-terminus (containing the tandem helix-turn-helix) also contributes to PA3027 dimerization (oligomerization). This suggests the possibility of the head to head or tail to tail, but also head to tail PA3027 self-interaction, similar to the one previously observed, for example, for the AraC regulator ExsA from *P*. *aeruginosa*, bound to the promoters of regulated genes (*exsC*, *exsD*, *exoT*, *pcrG*) [26].

AraC-type regulators can work as monomers (e.g., Rns in *E. coli*) [55], however, some need to form dimers (e.g., ExsA in *P. aeruginosa* [56] or oligomers, like UreR in *E. coli*) [57]. The AraC from *E. coli* acts as a dimer, however, dimerization and the mode of DNA binding are largely affected by the ligand [58]. The state of oligomerization of PA3027 and the mode of action could also be regulated by the presence of the ligand, however, additional studies are required for its identification. Remarkably, PA3027 readily activated the *PA3026*p in heterologous host *E*. *coli*, which suggests that either a specific ligand is not required for activation or that the hypothetical ligand is present in *E*. *coli* cells, grown in L broth medium.

Comparison of transcriptomes of cells with a slight PA3027 overproduction and identification of PA3027 binding sites allowed defining the PA3027 regulon (Table 2). The ChIP-seq analysis pointed out 24 PA3027 binding sites, but summits of only six of these mapped to regions directly preceding genes. Similarly, among the 28 binding sites of *P*. *aeruginosa* CdpR, only three were located in intergenic regions including the divergently transcribed promoter of *P. aeruginosa* quinolone signal (PQS) [29]. Remarkably, some of the PA3027 ChIP-seq peaks had complex shapes, and sometimes clear sub-summits, suggesting PA3027 binding to multiple motifs in the analyzed regions. The most significant observation of this work was the direct binding of PA3027 to the promoters of *PA3026*–*PA3023*, *PA3464*, and *PA3342* genes, also showing the spectacular increase of mRNA level in response to PA3027 excess, which indicates the role of PA3027 in the activation of these genes via direct interactions with their promoter regions. Our data show that this activation can be readily observed for *PA3026*p.

Our analysis identified two putative PA3027 binding motifs, motif A (15 bp, consensus YYGGCGHTDTYSGMC) and motif B (11 bp, consensus GGAYAWCGCCG) (Figure 3C,D). Similar to other AraC-type regulators (e.g., AraC from *E. coli* or CuxR from *Sinorhizobium meliloti*), PA3027 binds to more than one motif [59,60]. The identified PA3027 binding sites showed some similarity to the motifs recognized and bound by other AraC representatives (e.g., VqsM (GGATSNNNTYGGCCA) or CdpR (RGWYNNNWNCGGCCA) from *P. aeruginosa*) [5]. The localization of PA3027 binding motifs within promoter regions of activated genes, upstream or overlapping the −35 promoter region position (Figure 4A–C), was similar to those observed previously for binding sites of transcriptional activators (e.g., AraC, MarA) [9,37]. A careful analysis of *PA3026*p also indicated the presence of partial palindromes CCGGCGTGCGTGCCGG and GGCCGGCGGCGGCC as well as inverted repeat TCGGCCTGGA-N29-TCCAGGCCGA (Figure 4A), not directly resembling the PA3027 binding consensus for motif A or B, however, we might not rule out the involvement of these sequences in PA3027 action. The identified motifs are characterized by repeated stretches of GC pairs. The regulatory experiments and EMSA assays using truncated regions of the *PA3026* promoter suggested that to act efficiently in transcription activation, PA3027 needs multiple sites, one in the vicinity of the −35 promoter sequence and at least one more located upstream, at a distance. The shortest tested fragment of *PA3026*p, with one predicted PA3027 binding motif, exhibited the lowest increase in expression in response to PA3027, however, in EMSA analysis, this fragment was still bound by PA3027 (Figure 4F). Other tested variants of the *PA3026*p with more than one putative PA3027 binding motif were bound by PA3027 in EMSA and activated in response to PA3027 in the regulatory assays (Figure 4E). The presence of distal sites provides the possibility of modulating the action of the regulator in the presence or absence of a ligand, similar to the AraC regulator [9]; however, further studies are required to analyze the role of specific motifs in *PA3026*p activation by PA3027.

A spectacular increase of expression under PA3027 overproduction conditions was also observed for the *PA3464* gene encoding PlcA, a phospholipase C acting on phosphatidylcholine (PC), phosphatidylserine (PS), and phosphatidylethanolamine (PE) [61]. Divergently transcribed *PA3465* gene encoding conserved hypothetical protein classified as the membrane MSF (major facilitator superfamily domain) transporter was downregulated. Interestingly, according to ChIP-seq data, there were two PA3027 binding regions upstream and downstream of the *PA3464* gene (Figure A2). In *PA3464*p, motif A is located 23 bp upstream to the −35 sequence of *PA3464*p, while motif B overlaps it. The PA3027 binding sites in the promoter and terminator regions were also observed for the *PA3342* gene encoding a protein with an uncharacterized DUF2804 domain (Figure 3B). Only motif A located 133 bp to the start codon was identified in *PA3342*p. The cloned fragments preceding *PA3464* or *PA3342* were however not sufficient to promote PA3027 dependent gene activation in *E*. *coli*. Potentially like Rns, CfaR, VirF, AggR, and CsvR [62,63], PA3027 needs to have a second binding motif downstream of the promoter binding site to activate expression. In this case, the second motif would be in the gene terminator. These analyses point out that other genetic/proteic components are needed for the PA3027 dependent activation of these genes.

The *PA3026*–*PA3024* operon potentially encodes proteins involved in glycerolipid metabolism. *PA3024* encodes a putative FGGY carbohydrate kinase carrying out ATP-dependent phosphorylation of glycerol (Figure 5). Three other enzymes with this activity are encoded in the PAO1 genome: GlpK, PA1487, and PA3579, but only one, GlpK, has been characterized so far as a part of the GlpR regulon together with GlpDFK [64]. The GlpD homolog, PA3025, potentially catalyzes the next step in the pathway, oxidizing glycerol-3-phosphate to dihydroxyacetone phosphate (DHAP). *E. coli* encodes two glycerol 3-phosphate dehydrogenases, the *glpD* and *glpABC* genes. GlpD is required for aerobic growth with glycerol or glycerol 3-phosphate, while GlpABC is required for anaerobic growth with glycerol (or glycerol 3-phosphate) and fumarate [65,66]. *PA3026* encodes a potential oxidoreductase acting on the CH–OH group of donors. The next gene, *PA3023*, transcribed in the same orientation as the *PA3024*, encodes a probable diacylglycerol kinase, similar to the lipid kinase YegS from *Salmonella typhimurium* [67].

The ability to generate the Δ*PA3027* and Δ*PA3026*–*PA3024* strains indicates that the genes are not crucial for the growth of *P. aeruginosa*. The ability of the strains to grow in minimal media with different carbon sources (e.g., citrate, glycerol) was tested as several bacterial AraC transcription factors are known to control genes that are responsible for the degradation of complex carbon sources [68]. We cannot rule out that these genes play a role in the metabolism of glycerol and the fact that the Δ*PA3026*–*PA3024* operon mutant could grow on glycerol might be explained by the functional redundancy of the *glpD* homologs in *P. aeruginosa* [69]. Another gene that is potentially regulated directly by PA3027 is *PA3464*, which encodes a phospholipase C, cleaving phospholipids just before the phosphate group [70]. PA3464 is active on PC, PS, and PE [61], however, further studies are needed to elucidate the role of this enzyme in *P*. *aeruginosa* biology.

Engagement of the products of the regulated genes in glycerol metabolism could be a first step in the production of glycerolipids [66], biosurfactants [71], biofilm formation [72], or as an energy source [73]. The link between PA3027 target genes (*PA3026*–*PA3024*, *PA3464*) is phospholipid metabolism (Figure 5). Phospholipids are membrane components that could contain sn-glycerol-3-phosphate esterified with fatty acids [74]. The PE, phosphatidylglycerol, and cardiolipin are the major phospholipids in bacteria. All membrane phospholipids include phosphatidic acid (PA), which is derived from glycerol-3-P via action of glycerol-3-P acyltransferases [75]. The enzymes that catalyze the turnover of phospholipids include both phospholipases and lipid phosphatases. It is important to elucidate the complex regulatory mechanisms that control the connected and coordinated pathways involved in the synthesis of glycerophospholipids as important components of each cell. The work presented here is the first characterization of the PA3027 transcriptional regulator involved in the regulation of glycerolipid metabolism in *P. aeruginosa*, thus we propose the name GliR (glycerolipid metabolism regulator) for PA3027.

## 4. Materials and Methods

### 4.1. Growth Conditions Bacterial Strains and Plasmids

Bacterial strains, plasmids, and primers used in this study are listed in Table A2 and Table A3. *E. coli* and *P. aeruginosa* strains were grown at 37 °C in Luria-Bertani (LB) broth or on LB plates containing 1.5% (*w*/*v*) agar [76] as well as in M9 minimal medium [77] supplemented with 0.25% citrate ions or 1% glycerol as a carbon source and leucine (10 mM) for PAO1161 *leu*^−^ strain. For the selection of plasmids, LB medium was supplemented with appropriate antibiotics: kanamycin (50 μg mL^−1^ for *E. coli*, 500 μg mL^−1^ in solid media, and 250 μg mL^−1^ in liquid media for *P. aeruginosa*); benzylpenicillin sodium salt (300 μg mL^−1^ in solid media and 150 μg mL^−1^ in liquid media for *E. coli*); carbenicillin (300 μg mL^−1^ for *P. aeruginosa*); rifampicin (300 μg mL^−1^ for *P. aeruginosa*); and chloramphenicol (10 μg mL^−1^ for *E. coli*, 150 μg mL^−1^ for *P. aeruginosa*).

Bacterial cells were routinely grown in flasks closed with a cotton plug, with shaking 200 rpm at 37 °C. Growth kinetics was monitored by measurements of optical density at 600 nm (OD_600_) in 96-well plates at 37 °C using a Varioskan Lux Multimode Microplate Reader and SkanIt RE 5.0 software (Thermo Fisher Scientific, Waltham, MA, USA). Motility assays were performed as described previously [33,78]. Plates were standardized by using the same volume of medium. Biofilm analysis was performed according to the previously described method [77] using cultures in LB or minimal medium with citrate grown at 37 °C for 24 h or 48 h.

Competent cells of *E. coli* were prepared by the CaCl_2_ method [77] and *P*. *aeruginosa* according to the method using MgCl_2_ [79]. *E. coli* strain DH5α was used for plasmid manipulations and S17-1 was used to mate pAKE600 [80] derivatives into *P. aeruginosa*. Standard DNA manipulations were performed as described [77].

### 4.2. Vectors and Strains Construction

The Δ*PA3027* or Δ*PA3026*–*PA3024* deletion mutants were constructed with the use of pAKE600 suicide vector derivatives [80]: pKKB1.61 and pKKB1.62 (Table A2). Upstream and downstream DNA fragments of mutated regions were amplified using primer pairs #1/#2 and #3/#4 for *PA3027* or #5/#6 and #7/#8 for *PA3026*–*PA3024* (Table A3). The PCR fragments were digested with BamHI, HindIII, and HindIII, EcoRI, respectively, and ligated with EcoRI, BamHI digested pAKE600. The allele exchange procedure was performed as described previously [33]. The *E. coli* S17 strain carrying suicide plasmid was conjugated with the recipient strain *P. aeruginosa* PAO1161 (Rif^R^). Putative cointegrants were selected on LB agar with rifampicin and carbenicillin. Colonies were used to inoculate LB with 10% sucrose and checked for the Cb^s^ phenotype to select clones without the vector. Allele exchange was screened by PCR (Table A3).

The *PA3027* gene was cloned in pET28a to obtain the His_6_–tagged version of the protein at the N-terminus. The gene was amplified using PCR with primers #9/#10 and PAO1161 genomic DNA as a template. The purified fragment was then digested with EcoRI, SacI and ligated with EcoRI, SacI digested pET28a to obtain pKKB1.21.

To obtain an expression vector able to propagate in *P. aeruginosa* (pKKB1.11 and pKKB1.12), the *PA3027* gene was excised from pKKB1.21 using EcoRI, SalI and inserted into pAMB9.37 or pABB28.1, a derivative of pBBR1MCS-1 [81,82], containing *tac*p and *lacI*^Q^ or *lacI^Q^*, *tac*p, and *flag*, respectively.

To test the promoter activity of selected DNA fragments, the RK2 derivative pCM132 plasmid with a promoter-less *lacZ* reporter gene was used [47]. PCR products corresponding to *PA3026*pA (120 bp), amplified using primers #14/#13), *PA3026*pB (176 bp amplified using primers #12/#13), *PA3026*pC (330 bp, amplified using primers #15/#13), *PA3342*p (167 bp, amplified using primers #18/#19), were digested with EcoRI, BamHI, and ligated with EcoRI, BglII digested pCM132 to obtain pKKB1.305, pKKB1.303, pKKB1.304, and pKKB1.309, respectively (Table A2). pKKB1.307 (*PA3464*p-*lacZ*) was constructed by amplification of the 228 bp fragment using primers #16/#17, followed by digestion with BglII and ligation with BglII digested pCM132. The scheme of cloned and analyzed variants of the *PA3026* promoter region is shown in Figure 4.

For bacterial two-hybrid analysis, DNA fragment encoding PA3027 was cloned into derivatives of pKT25 (pLKB2), pKNT25, pUT18, and pUT18C (pLKB4) [83]. To obtain pKKB1.51 and pKKB1.81 (pKNT25, pUT18 derivatives, respectively with *PA3027* lacking STOP codon), the PCR fragments amplified using #9/#11 primers were digested with EcoRI, SacI and ligated with vectors digested with the same enzymes (Table A2). To construct pKKB1.52, *PA3027* was excised from pKKB1.11 using EcoRI/SmaI and ligated with pLKB2 digested with EcoRI, Ecl136II. The *PA3027* gene was transferred from pKKB1.21 to pLKB4 as an EcoRI, SacI fragment to obtain pKKB1.82 (Table A2).

### 4.3. Protein Overproduction and Purification

Overproduction of His_6_-PA3027 was carried out in the *E. coli* BL21 strain carrying pKKB1.21. Overnight culture was diluted 1:50 in 1000 mL autoinduction LB (Formedium, Norfolk, UK) supplemented with 1% glycerol, 0.5% NaCl, and kanamycin and grown for 48 h at 18 °C. Cells were harvested by centrifugation and sonicated in Buffer S (20 mM MES pH 6.5, 200 mM (NH_4_)_2_SO_4_, 200 mM NaCl) with 1 mM phenylmethylsulfonyl fluoride (PMSF) and 1 mg/mL lysozyme. The His_6_-tagged PA3027 was purified on Ni-agarose columns (Ni-TED 1000 Protino, Marchel&Nagel) using Buffer S with 250 mM imidazole for elution. The purification procedure was monitored by sodium dodecyl sulfate polyacrylamide gel electrophoresis (SDS-PAGE) using a Pharmacia PHAST gel system. Elution fractions were dialyzed using Buffer S with 1% β-mercaptoethanol and stored at −80 °C.

### 4.4. SEC-MALS Analysis

Size exclusion chromatography coupled to multi-angle light scattering (SEC-MALS) analysis was performed using a high-performance liquid chromatography (HPLC) instrument (1260 Infinity LC, Agilent Technologies Inc., Santa Clara, CA, USA) equipped with a UV detector, a MALS detector (DAWN HELEOS II, Wyatt Technology Santa Barbara, CA, USA), and a differential refractometer (Optilab T-rEX, Wyatt Technology, Santa Barbara, CA, USA). A total of 100 µL of 1 mg/mL samples, obtained as described in the above section, were loaded onto a Superdex 200 Increase 10/300 column (GE Healthcare, Milwaukee, WI, USA) equilibrated with Buffer S. Absorption at UV wavelengths of 280, 254, and 215 nm were monitored during SEC. Samples were run at room temperature at a flow rate of 0.5 mL/min. The results were analyzed using ASTRA v. 6 software (Wyatt Technology, Santa Barbara, CA, USA) in accordance with the manufacturer’s instructions.

### 4.5. Glutaraldehyde Crosslinking

The oligomerization state of purified His_6_–PA3027 was assayed by crosslinking as described previously [84].

### 4.6. RNA Isolation, RNA-Seq, and RT-qPCR Analysis

Strains were obtained by transformation of PAO1161 cells with pKKB1.11 (*tac*p–*PA3027*) or pAMB9.37 (*tac*p) plasmids (Table A2). Transformants were selected on L-agar plates supplemented with 150 μg mL^−1^ chloramphenicol and were verified by isolation of plasmid DNA and its digestion. After overnight growth, each culture of *P. aeruginosa* PAO1161 carrying pKKB1.11 or pAMB9.37 vector was diluted 1:100 into fresh L-broth supplemented with 75 μg mL^−1^ chloramphenicol and 0.05 mM IPTG as inducer. Cells were collected from 2 mL of cultures in the logarithmic phase of growth (optical density at 600 nm of 0.4–0.6) were mixed with 4 mL of RNAprotect Bacteria Reagent (Qiagen, Hilden, Germany). RNA was isolated using the Qiagen RNeasy Mini Kit, according to the manufacturer’s instructions. RNA was treated with DNA-free DNA Removal Kit (Invitrogen, Thermo Fisher Scientific, Waltham, MA, USA) and a lack of DNA contamination was confirmed by PCR. RNA concentration was determined using µDrop plate of Varioskan Lux Multimode Microplate Reader and quality was checked using Bioanalyzer.

Library preparation and sequencing were performed at the Laboratory of DNA Sequencing and Oligonucleotide Synthesis, Institute of Biochemistry and Biophysics, PAS (Warsaw, Poland). Ribosomal RNA was depleted using the RiboZero Bacteria Kit (Illumina, San Diego, CA, USA). Obtained mRNA was used for cDNA library construction using the KAPA Stranded RNASeq Kit. The library was further quality checked on 1% agarose gel and concentration was measured using the qPCR KAPA Library Quantification Kit (Roche Holding AG, Basel, Switzerland). Libraries were sequenced using standard Illumina protocols. Reads were quality-checked and filtered using FASTP version 0.20.0 [85]. Reads were mapped to the *P*. *aeruginosa* PAO1161 genome (CP032126.1) using Bowtie2 version 2.3.4.3 [86] with default settings. The number of reads mapping to individual genes was counted using FeatureCounts v 2.0.1 (part of the Subread) with the -s2 option [87]. Differential expression analysis was conducted using edgeR ver 3.28.0 [88]. Raw data are available in the NCBI’s Gene Expression Omnibus (GEO) database under accession number GSE163555.

For selected genes, the results were confirmed by RT-qPCR using RNA isolated from the same cultures. Reverse transcription on 4 µg RNA was performed using a TranScriba Kit (A&A Biotechnology, Gdansk, Poland). qPCR was performed on a LightCycler 480 II System (Roche Molecular Diagnostics, Mannheim, Germany) using 5× HOT FIREPol EvaGreen qPCR Mix Plus (Solis Biodyne, Tartu, Estonia). Each 18 µL reaction contained 3.6 µL 5× reaction mix, 1 µL of five times diluted cDNA and 1.5 µL of mixed 5 µM primers (Table A3). The relative expression was determined by comparison of crossing points (Cp) between the target and the reference gene (*nadB*). Three technical repetitions were used for each primer pair. The ratio was calculated using the Pfaffl formula [89].

### 4.7. Chromatin Immunoprecipitation with Sequencing

ChIP-seq analysis was performed using the PAO1161 Δ*PA3027* strain with pKKB1.12 (strain overproducing FLAG–PA3027) and PAO1161 Δ*PA3027* with pABB28.1 (*lacI^Q^*–*tacp*–*flag*). Cells were grown at 37 °C in LB with 50 μg mL^−1^ chloramphenicol and 0.05 mM IPTG. Cells were collected at OD_600_ of 0.5. ChIP protocol was based on a modified protocol using Dynabeads Protein A [90]. Lysate after sonication was thawed on ice and 150 μL of each strain variant was incubated with 20 μL of magnetic beads coupled with protein A (Dynabeads Protein A, 10001D, Invitrogen, Thermo Fisher Scientific, Waltham, MA, USA), separated from the original suspension using a magnetic separation stand. A pre-clearing step was performed for 1 h at 4 °C with rotation. A total of 50 μL of magnetic beads, separated from the suspension as above, was then mixed with 6 μL of anti-FLAG mouse polyclonal antibodies (DYKDDDDK Tag polyclonal antibodies 1 mg/mL, PA1-985B, Invitrogen, Thermofisher Scientific, Waltham, MA, USA) diluted in 200 μL of PBS with 0.05% Tween-20. Mixtures of magnetic beads and antibodies were incubated for 10 min at 4 °C with gentle rotation. Beads with bound antibodies were then separated from the supernatant, washed once with 200 μL of the PBS with 0.05% Tween-20 solution, and stored on ice. Pre-cleared lysate was separated from the beads used for pre-clearing and added to the beads coated with antibodies. A mixture containing lysate and magnetic beads with antibodies was incubated at 4 °C for 20 min with mixing on a rotator. Beads were then collected and washed as described earlier [35]. Elution was performed twice for 15 min in 50 μL at 65 °C in a thermoblock (Thermomixer compact, Eppendorf, Hamburg, Germany) with 1400 rpm (7× *g*) mixing. Elutions from six parallel reactions were pooled. The obtained eluates were incubated with 8 μL of RNase A (100 mg/mL, 19101, Qiagen, Hilden, Germany) for 30 min at 65 °C. Then, 40 μL of Proteinase K (20 mg/mL, 19133, Qiagen, Hilden, Germany) was added and the samples were incubated for 1 h at 50 °C, followed by overnight incubation at 65 °C. Next, 40 μL of Proteinase K was added and the samples were again incubated for 1 h at 50 °C. Subsequently 24 μL of 3 M sodium acetate (pH = 5) was added and the volume was adjusted to 700 μL using water. DNA purification was performed using a Qiaquick Qiagen PCR purification Kit, according to the manufacturer’s instructions. The DNA was stored at −20 °C. Purified DNA from ChIP performed with the empty vector strain was included as a background control.

Sequencing of ChIP samples was performed in the Laboratory of DNA Sequencing and Oligonucleotide Synthesis of Institute of Biochemistry and Biophysics Polish Academy of Sciences in Warsaw, Poland. The NGS library was constructed using a QiaSeq Ultralow Input Library Kit (Qiagen, Hilden, Germany). Samples were quality checked on 1% agarose gel and concentration was measured using a qPCR KAPA Library Quantification Kit (Roche Holding AG, Basel, Switzerland). Libraries were sequenced using standard Illumina protocols.

Reads were quality-checked and filtered using FASTP version 0.20.0 [85]. Reads were mapped to the *P*. *aeruginosa* PAO11161 genome (CP032126.1) using Bowtie2 version 2.3.4.3 [86] using default settings. Obtained *.sam files were sorted (samtools sort -*n*), run through samtools fixmate with the -m option, again sorted (samtools sort), and duplicates were marked with samtools markdup. Samtools ver. 1.9 was used [91]. The files were indexed and used to generate coverage *.bigwig files, normalized to 1× sequencing depth (RPGC), without binning and smoothing using the bamCoverage tool ver 3.3.0 included in deepTools [92].

ChIP-seq peaks were called separately for each ChIP sample using MACS2 ver 2.1.2 [93] with default options for paired-end BAM files, and 0.05 as the false discovery rate (FDR). Subsequently, peak matching was performed using the findOverlapsOfPeaks function from ChIPpeakAnno [94]. Peaks overlapping and regions enriched in the background control were excluded. Visualization of the coverage data was performed using Integrated Genomics Viewer ver 2.4.17 [95]. Peaks were annotated using a custom R script. Raw data are available in the NCBI’s Gene Expression Omnibus (GEO) database under accession number GSE163554.

### 4.8. Regulatory Experiments

The β-galactosidase activity was tested in extracts from exponentially growing *E. coli* DH5αΔ*lac* cells bearing pCM132 derivatives (pKKB1.305 *PA3026*pA, pKKB1.303 *PA3026*pB, pKKB1.304 *PA3026*pC, pKKB1.309 *PA3342*p, pKKB1.307 *PA3464*p), and pKKB1.11 (*tac*p–*PA3027*) or pAMB9.37. To assay β-galactosidase activity, bacteria were grown at 37 °C in L-broth containing kanamycin and chloramphenicol with or without 0.1 mM IPTG. The β-galactosidase activity was calculated using the J. Keith Joung modified Miler Units equation [96].

### 4.9. Electrophoretic Mobility Shift Assay (EMSA)

The electrophoretic mobility shift assay (EMSA) was performed using purified His_6_-PA3027 and the amplified, purified *PA3026* promoter variants (Figure 4). To prepare DNA fragments, PCR was performed using pCM132 derivatives as a template and appropriate pair of primers as follows: pKKB1.305 for the *PA3026*pA fragment (#14/#20), pKKB1.303 for the *PA3026*pB fragment (#12/#20), pKKB1.304 for the *PA3026*pC fragment (#15/#20), pKKB1.309 for the *PA3342*p fragment (#18/#20), pKKB1.307 for the *PA3464*p fragment (#16/#20), and pCM132 for the control fragment (#21/#20). The reverse primer (#20), which binds to the plasmid sequence was coupled with Cy5 dye, enabling visualization using a FluorChemQ MultiImageII ChemiImager. The images were captured using AlphaView software (Alpha Innotech, version 3.5.0). The binding reaction was performed in the presence of unspecific DNA (600 ng, salmon sperm DNA) and the complexes were analyzed on a 10% acrylamide gel.

### 4.10. Bacterial Two-Hybrid Analysis (BATCH)

Appropriate pairs of plasmids were co-transformed into the *E. coli* BTH101 *cya^-^* strain [83]. The presence of interactions between the tested proteins was assayed by the analysis of the appearance of red colonies of transformants on the MacConkey medium and β-galactosidase activity in the extracts of transformants [96].

### 4.11. Bioinformatic Analysis

A comparison of the C-terminal domain of the chosen AraC-type regulators was performed using Clustal W. Structure of the PA3027 monomer bound with DNA was predicted using COACH and HDOCK [38,39,40]. Clustered orthologs of PA3024-PA3027 were identified in 1748 reference genomes from the Refseq database (Release 91) using MultiGeneBlast [97]. The DNA binding motifs (Table A1) were identified using MEME-ChIP version 5.3.0 [44,45] using sequences corresponding to 200 bp around 24 PA3027 peak summits (Supplementary Text S1).

## 5. Conclusions

The AraC-type regulators are involved in the control of various cellular functions, helping to adjust metabolism to efficiently use available recourses, maintain homeostasis, and propagate. Here, we performed a thorough analysis of a representative of the AraC family PA3027 transcriptional regulator from *P*. *aeruginosa*. The regulon of PA3027 was identified, highlighting the role of PA3027, named GliR in the regulation of genes involved in glycerolipid metabolism. The significance of our research is in identifying the pathways regulated by GliR, which in turn allows for a better understanding of the complicated regulatory network of the human pathogen *P. aeruginosa*.

## Figures and Tables

**Table 1:** Genes with altered expression in response to PA3027 excess (fold change <−10 or >10). PseudoCAP categories description as in Figure 1A.

PAO1 ID	PAO1161 ID (D3C65_)	Fold Change in RNA-seq	PseudoCAP Category	Gene Product
PA3024	10155	173.40	EM; PE	carbohydrate kinase
PA3025	10150	163.31	CCC; EM	glycerol-3-phosphate dehydrogenase/oxidase
PA3026	10145	135.03	HUU	FAD-binding oxidoreductase
PA3464	07800	129.57	HUU	phospholipase
PA3027	10140	107.00	TR	AraC family transcriptional regulator
PA3023	10160	47.32	HUU	lipid kinase YegS
PA2202	14525	15.56	MP; TSM	amino acid ABC transporter permease
PA3342	08435	15.38	MP	DUF2804 domain-containing protein
PA2203	14520	14.14	MP; TSM	amino acid ABC transporter permease
PA5024	27340	13.07	HUU	sulfite exporter TauE/SafE family protein
PA4191	04005	13.06	PE	isopenicillin N synthase family oxygenase
PA4193	03995	12.57	MP; TSM	amino acid ABC transporter permease
PA4192	04000	10.44	TSM	amino acid ABC transporter ATP-binding protein
PA0282	01495	10.01	MP; TSM	sulfate ABC transporter permease subunit CysT
PA3392	08180	−11.34	EM	TAT-dependent nitrous-oxide reductase
PA0519	02740	−11.50	EM	nitrite reductase
PA0524	02765	−23.76	EM	nitric-oxide reductase large subunit
PA3391	08185	−44.43	EM; MP	regulatory protein NosR
PA0523	02760	−62.05	EM	cytochrome c

**Table 2:** *P. aeruginosa* loci with PA3027 binding sites identified in the ChIP-seq analysis.

Region ID	Binding Site Position	Gene in PAO1161 (D3C65_)	Gene in PAO1	Fold Change (RNA-seq)	Fold Enrichment (ChIP-seq)	Position of Summit	PseudoCAP Category	Gene Product
1	term	01245	PA0234	−1.71	5.73	264,723	HUU	nucleoside-binding protein
2	term	04760	PA4045	1.54	3.76	987,296	HUU	cobalamin-binding protein
3	intra	07240	PA3572	−2.18	7.06	1,510,957	HUU	hypothetical protein
4	prom	07800	PA3464	129.57			HUU	phospholipase
		07795	PA3465	−3.80			MP	conserved hypothetical protein
5	term	07800	PA3464	129.57	11.84	1,641,558	HUU	phospholipase
6	prom	08155	PA3397	1.38	3.72	1,712,386	BCPGC; EM	ferredoxin-NADP reductase
		08150	PA3398	1.17			TR	probable transcriptional regulator
7	intra	08185	PA3391	−44.43	4.30	1,720,088	EM; MP	regulatory protein NosR
8	prom	08435	PA3342	15.38	5.68	1,762,086	MP	DUF2804 domain-containing protein
9	intra	08440	PA3341	−1.16	2.42	1,763,471	TR	MarR family transcriptional regulator
10	intra	08605	PA3309	−3.36	9.83	1,805,406	HUU	universal stress protein
11	intra	09845	PA3083	1.51	8.26	2,055,622	TPTMD	aminopeptidase
12	intra	11,685	PA2742	−1.20	3.10	2,411,617	TPTMD	50S ribosomal protein L35
13	prom	12,415	PA2601	1.04	3.90	2,570,649	TR	LysR family transcriptional regulator
		12,420	PA2602	1.75			HUU	3-mercaptopropionate dioxygenase
14	term	12,415	PA2601	1.04			TR	LysR family transcriptional regulator
15	intra	14,830	PA2147	−1.73	5.28	3,153,105	AP	catalase HPII
16	intra	18,720	PA1414	−2.19	9.03	3,976,577	HUU	hypothetical protein
17	prom	19,855	PA1196	−4.91	3.16	4,218,075	TR	sigma-54-dependent Fis family transcriptional regulator
		19,860	PA1195	1.35			HUU	dimethylarginine dimethylaminohydrolase DdaH
18	intra	21,295	PA0928	−1.04	4.07	4,499,850	TCRS	hybrid sensor histidine kinase/response regulator
19	intra	25,030	PA4610	−3.03	2.56	5,276,490	HUU	copper transporter
20	term	26,025	PA4772	1.83	3.09	5,480,812	EM	FAD-binding oxidoreductase
21	intra	28,310	PA5208	−3.54	3.28	5,982,827	HUU	TIGR00153 family protein
22	intra	28,490	PA5243	−1.56	3.49	6,024,153	BCPGC	porphobilinogen synthase
23	prom	28,760	PA5294	−1.60	3.02	6,078,786	HUU	multidrug resistance protein NorM
		28,755	PA5293	−1.17			TR	probable transcriptional regulator
24	prom	10,145	PA3026	135.03	1.89	2,124,729	HUU	FAD-binding oxidoreductase

PA3027 ChIP-seq peaks identified in promoters (prom), terminators (term), or gene body (intra). Underlined regions (4 and 14) are second peaks in regions encompassing PA3027 binding sites with two clearly separated signals. PseudoCAP category descriptions as in Figure 1A.

## Data Availability

The raw RNA-seq and ChIP-seq data supporting the results of this article were deposited in the NCBI’s Gene Expression Omnibus (GEO) database (http://www.ncbi.nlm.nih.gov/geo/, 19 December 2020) and is accessible through GEO Series accession numbers GSE163555 and GSE163554.

## Supplementary Materials

The following are available online at https://www.mdpi.com/article/10.3390/ijms22105066/s1, Text S1: Sequences of PA3027 ChIP-seq peak summits used for motif searching (peaks marked by numbers and gene ID identified in the vicinity, see Table 2). Table S1: Results of RNA-seq analysis. List of genes with altered expression identified by comparison of transcriptomes of *P. aeruginosa* PAO1161 cells overproducing PA3027 with transcriptomes of cells carrying the empty vector (fold change (FC) ≤ −2 or ≥ 2, FDR adjusted *p*-value ≤ 0.05). Genes annotated only in PAO1161 strain but not in PAO1 are described as “not annotated” (NA). Table S2: Results of ChIP-seq analysis. PA3027-FLAG ChIP-seq peaks identified in each repeat—set A, B, C or in the negative control—D (Table S2A–D). Table S3: Distribution and evolutionary conservation of *PA3027-PA3023* cluster in bacteria identified using MultiGeneBlast [97].

## Appendix A

**Table A1:** PA3027 DNA binding motifs in ChIP-seq peaks. The DNA binding motifs were identified using MEME-ChIP version 5.3.0 [44,45] and DNA corresponding to 200 bp around 24 summits of ChIP-seq peaks (Supplementary Text S1). Motif A was identified with the settings, allowing one occurrence per sequence, whereas motif B was searched using a setting allowing zero or one occurrence per sequence.

Peak Number	Strand	Start	*p*-Value	Site
Motif A				
20	+	71	9.60 × 10^−8^	CTGGCGCTATTCGCC
2	+	72	5.89 × 10^−7^	GTGGCGTTATTCGCC
1	+	10	2.01 × 10^−6^	CCGGCGTTATCCGCC
9	+	47	5.80 × 10^−6^	TTGGTGTTGTTGCAT
7	+	37	5.80 × 10^−6^	CTGGCGCAGTTGGAC
5	+	2	7.42 × 10^−6^	CGGGCGTTATCCGCC
15	+	34	1.32 × 10^−5^	ATGGCGCGATCGGAC
11	+	40	1.32 × 10^−5^	CCGGCGAAATTCGCC
12	−	28	1.48 × 10^−5^	TTGGCATGTTCGGAT
21	+	60	2.78 × 10^−5^	TTGGCGACTTTGTCC
16	+	22	2.78 × 10^−5^	CCGGCGTTGCTCGTC
4	+	36	4.53 × 10^−5^	ATGGCGCTATCGGAG
17	−	23	7.14 × 10^−5^	GCGCCGATTTTCGAC
10	+	4	7.14 × 10^−5^	TCGACGTTGCTCGCC
23	−	62	7.79 × 10^−5^	CTGGCATGGCTCCAC
18	+	14	7.79 × 10^−5^	CTGGCGCCGCTGGCC
24	−	17	7.79 × 10^−5^	CTGTCACTGTCGGAT
8	+	39	1.62 × 10^−4^	TGGCCGATTTTGTCC
13	−	44	2.03 × 10^−4^	ATTGCATTTCTCGCA
19	+	54	2.18 × 10^−4^	TCGTCGCGGTCGGAC
3	−	83	2.35 × 10^−4^	CTGGCAAGTCTGCCT
22	−	16	3.31 × 10^−4^	TCGGTGATCCTCGAA
6	+	9	4.03 × 10^−4^	ATCGTGTTATCCCAT
14	−	70	4.29 × 10^−4^	TGGGTGCTTTTCTTA
**Motif B**				
2	+	61	7.20 × 10^−7^	GGATAACGCCG
1	−	11	7.20 × 10^−7^	GGATAACGCCG
21	−	40	1.90 × 10^−6^	GGACATCGCCG
20	+	60	3.50 × 10^−6^	GGATAGCGCCG
5	−	3	4.83 × 10^−6^	GGATAACGCCC
24	−	43	6.79 × 10^−6^	GGACATCGCCC
16	−	33	1.22 × 10^−5^	GGAAAACGACG
18	+	49	1.48 × 10^−5^	GGATACCGCCG
17	+	77	1.84 × 10^−5^	GGAAATCGTCG
13	+	83	2.31 × 10^−5^	TGACATCGACG
7	+	24	3.02 × 10^−5^	TGATGTCGCCG
19	+	19	3.88 × 10^−5^	GCACATCGCCG
22	+	51	7.02 × 10^−5^	GGATGGCGCCC
4	+	69	8.23 × 10^−5^	GGACAGTGCCC
3	+	60	9.79 × 10^−5^	GGAAGTTGACG

**Table A2:** Bacterial strains and plasmids used and constructed in this study.

Strains		
*E. coli* DH5α	F^−^ (φ80d *lacZ*ΔM15) *recA1 endA1 gyrA96 thi-1 hsdR17* (r_k_^−^m_k_^+^) *supE44 relA1 deoR* Δ(*lacZYA*–argF)U196	[100]
*E. coli* BL21	F^−^ *ompT hsdS* (r_B_^−^m_B_^−^) *gal dcm* (*λ* DE3)	[100]
*E. coli* S17-1	*pro hsdR hsdM recA* Tp^R^ Sm^R^ΩRP4-Tc::Mu-Km::Tn*7*	[100]
*E. coli* BTH101	F^−^cya^−99^ *araD139 galE15 galK16 rpsL1* (Str^r^) *hsdR2 mcrA1 mcrB1*	[83]
*E. coli* DH5α Δ*lac*	F^−^ (φ80d *lacZ*ΔM15) *rec**A1 end**A1 gyr**A96 thi*-*1 hsd**R17* (r_k_^-^m_k_^+^) *sup**E44 rel**A1 deo*R Δ(*lacZYA*–*argF*)U196	lab collection
*P. aeruginosa* PAO1161	*leu* r^−^m^+^ Rif^R^	[101]
*P. aeruginosa* PAO1161 Δ*PA3027*	deletion of 1015 bp fragment encompassing *PA3027* gene	this work
*P. aeruginosa* PAO1161 Δ*PA3026*–*PA3024*	deletion of 4711 bp fragment encompassing *PA3026-PA3024* operon	this work
***Plasmids***		
pAKE600	Ap^R^; *ori*_MB1,_ *oriT*_RK2_, *sacB*	[80]
pKKB1.61	Ap^R^; pAKE600 derivative with 431 bp fragment encompassing up- and down- sequence of *PA3027*, cloned using BamHI-HindIII-EcoRI and 1#/2# and 3#/4# primers	this work
pKKB1.62	Ap^R^; pAKE600 derivative with 472bp fragment encompassing up- and down- sequence of *PA3026*–*PA3024*, cloned using BamHI, HindIII/HindIII, EcoRI EcoRI and 4#/5# and 6#/7# primers	this work
pET28a(+)	Km^R^; *ori*_pBR322_; *ori*_f1_; expression vector	Novagen
pKKB1.21	pET28a(+) derivative with *his*_6_–*PA3027*, PA3027 cloned 1078 bp as EcoRI, SacI	this work
pBBR1-MCS1	Cm^R^; IncA/C broad-host-range cloning vector, *lacZα*–MCS, *mob*, T7p, T3p	[81]
pAMB9.37	Cm^R^, *oriC*_IncA/C_, pBBR1-MCS-1 derivative with *lacI^Q^*–*tac*p, expression vector	[82]
pABB28.1	Cm^R^, *oriC*_IncA/C_, pBBR1-MCS-1 derivative with *lacI^Q^*–*tac*p–*flag* expression vector	this work
pKKB1.11	pAMB9.37 (*lacI^Q^*–*tac*p) derivative with 1097 bp fragment, encoding *PA3027* gene, cloned using EcoRI, SalI	this work
pKKB1.12	pABB28.1 (*lacI^Q^*–*tac*p–*flag*) derivative with 1097 bp *flag*–*PA3027* fragment cloned using EcoRI, SalI	this work
pLKB2	Km^R^, *ori*_p15_, pKT25 modified with *lac*p–*cyaT25*–MCS	[83,102]
pKNT25	Km^R^; *ori*_p15_, *lac*p–MCS–*cyaT25*,	[83]
pUT18	Ap^R^; *ori*_ColE1_, *lac*p–MCS–*cyaT18*,	[83]
pLKB4	Ap^R^, *ori*_ColE1_, pUT18C modified with*lac*p–*cyaT18*–MCS	[83,102]
pKKB1.51	pKNT25 derivative with *PA3027*–*cyaT25*; 1042 bp *PA3027* fragment cloned using EcoRI, SacI enzymes	this work
pKKB1.52	pLKB2 derivative with *cyaT25–PA30*27; 1092 bp *PA3027* fragment cloned using EcoRI, Ecl136II/SmaI enzymes	this work
pKKB1.81	pUT18 derivative with *PA3027*–*cyaT18*; 1042 bp *PA3027* fragment cloned using EcoRI, SacI enzymes	this work
pKKB1.82	pLKB4 derivative with *cyaT18*–*PA3027;* 1092 bp *PA3027* fragment cloned using EcoRI, SacI enzymes	this work
pCM132	Km^R^; *oriV*_RK2_; *oriT*_RK2_; promoter-less *lacZ* reporter gene	[47]
pKKB1.305	pCM132 derivative with *PA3026*pA, 120 bp fragment amplified with primers 14#/13# cloned using EcoRI, BamHI/BglII	this work
pKKB1.303	pCM132 derivative with *PA3026*pB, 176 bp fragment amplified with primers 12#/13# cloned using EcoRI, BamHI/BglII	this work
pKKB1.304	pCM132 derivative with *PA3026*pC, 330 bp fragment amplified with primers 15#/13# cloned using EcoRI, BamHI/BglII	this work
pKKB1.307	pCM132 derivative with *PA3464*p, 228 bp fragment amplified with primers 16#/17# cloned using BglII	this work
pKKB1.309	pCM132 derivative with *PA3342*p, 167 bp fragment amplified with primers 18#/19# cloned using EcoRI, BamHI/BglII	this work

MCS—multiple cloning site.

**Table A3:** List of primers used in this study.

Nr	Starter	Used for:	Sequence
#1	3027mLF	*PA3027* gene deletion	gcggatcCAATTCGACCACGGTGCTTTC
#2	3027mLR		gcaagcttGGTCTGCATGGTCGTTGTTC
#3	3027mPF		gcaagctttagtAATGAGAACGGCGGCCATCCG
#4	3027mPR		gcgaattcCGGTGCTCTATCCGAACCAGAGTTCC
#5	3026-4mLF	*PA3026*–*PA3024* operon deletion	gcggatccCATGGCGCAGGTATTTCAGC
#6	3026-4mLR		gcaagcttACGACGCATGAACCTCTCC
#7	3026-4mPF		gcaagctttagtaaTGAACCGGGCGCCGCACTTCC
#8	3026-4mPR		gcgaattcTCGCCGTCCTCCCAGGTTAC
#9	3027eF	*PA3027* expression	gcgaattcATGCAGACCCTTGGCTCCAC
#10	3027eR		gcgaGcTCAGCGAACTGCTCGATTG
#11	3027eR2		gcgagctcgTTGCGCCGCCGGCTCCTTGC
#12	3027pdF	*PA3026* promoter	cagaattcgcatgcGGTCGTTGTTCTTGTCGTGGCGGG
#13	p3026R2		caggatccGAACCTCTCCCCTCGCTTGT
#14	p3026F2		cagaattcgcatgcTCTTGATCGGTCACAGGGAG
#15	p3026F3		cagaattcgcatgcGGGCTTCGCCCGGCAGGCGC
#16	p3464R	*PA3464* promoter	gcagatctCGGCTTTTCGCTCTTGTCTC
#17	p3464F		gcagatctCAGATGGGTGCCTTGTATCA
#18	p3342F	*PA3342* promoter	cagaattcgcatgcGCAACGACCTGAAGCGTCTC
#19	p3342R		caggatccGTCCGTCCGGAAGTCGTCTC
#20	CM132RCy5	EMSA analysis	Cy5—CTTCCACAGTAGTTCACCACC
#21	CM132pF		GTGAACGCTCTCCTGAGTAG
#22	3027qF	RT-qPCR	CTGGATCGCCGACCTGGAAG
#23	3027qR		CCGGACAGCCGAAGAAGGTC
#24	3026qF		AATCGCTACCTTCCCGGCATCC
#25	3026qR		ATGATCCCGAAGCGTCCCTCAG
#26	3464qF		CAACCTGTTCAGCGACAACC
#27	3464qR		TAGAAGCCCATGTGGAACGG
#28	3023qF		CTACCTGTTGACCGGGTTGA
#29	3023qR		CCTTCCCACTGGAAGTCCG
#30	3342qF		GAGAACCGTATGGCTCACCG
#31	3342qR		TACACAGGCACATCGGCTG
#32	2203qF		TCTTCTGGTACTTCGGCGTT
#33	2203qR		GAGGAACTCGAAGGACGGC
#34	2204qF		CCCCTGCCAAACTCCCTTC
#35	2204qR		GATCAGGGCCTTGCAATGTG
#36	1911qF		AGATCTTCGTCGATAGCCGC
#37	1911qR		CATCTCCTGGCGTACGTTGA
#38	2398qF		CGACCAACCCCGCCATCACC
#39	2398qR		CCAGCCTGAACCTGCCAGCC
#40	3973qF		GGATCCTGAAGTCGACGAGC
#41	3973qR		GAAAGCTGGAATGCGCCAC
#42	2384qF		CGTCTCTCCGAAACCGGTAC
#43	2384qR		TGTAGACATCCTGTTCGTCGAG
#44	3465qF		CTGGTGATGATGCGCTCCTT
#45	3465qR		AGCATGTAGCCGTAGATCGC
#46	1196qF		TGGAAAGTACCCTGTTCGGC
#47	1196qR		ATCTCGTCGAGGAACAAGCTG
#48	2664qF		CCGAGGGATTGCTGAGCCGC
#49	2664qR		GCCATGAAAGGCCGGGGTCC
#50	nadBF		CTACCTGGACATCAGCCACA
#51	nadBR		GGTAATGTCGATGCCGAAGT
