# Supplementary material for: The AraC-Type Transcriptional Regulator GliR (PA3027) Activates Genes of Glycerolipid Metabolism in Pseudomonas aeruginosa
Source: Int J Mol Sci. 2021 May 11;22(10):5066. doi: 10.3390/ijms22105066 (PMC8151288; doi:10.3390/ijms22105066)
Supplement: Supplementary file 1 [file ijms-22-05066-s001.zip › Text S1.pdf]

The AraC-type transcriptional regulator GliR (PA3027) activates genes of glycerolipid metabolism in *Pseudomonas aeruginosa*

Karolina Kotecka, Adam Kawalek, Kamil Kobylecki, Aneta Agnieszka Bartosik*
Institute of Biochemistry and Biophysics, Polish Academy of Sciences, 02-106 Warsaw, Poland
*Correspondence: anetab2@ibb.waw.pl

Supplementary Materials

Text S1: Sequences of PA3027 ChIP-seq peak summits used for motif searching (peaks marked by numbers and gene ID identified in the vicinity, see Table 2).

```
>1 PA0235
CCGCGAGCGGTTATGCGCCCTACGCGATGCCGACCCCTGGGGTAGGGCGAATAACGCCACCGGCGTTATCCGCCG
ATACACCGAAGCCCGGCGGCGGATAACCGCAAGCGGTTATGCGCCCTACGCGATGCCGACCCCTGGGGTAGGGCG
AATAACGCCATCGGCGTTATCCGCCGATACACCGAAGCCCGGCGGCGGAT

>2 PA4045
TAACGCCGGTGGCGTTATTCGCCCTACGGCAATTGGGGGGTAGGGCGAATAACCGCAACGCGGTTATCGCTGCCA
GTCGGCGGGGCATGTCGGTATGGTGGTGTATCGGCGGATAACGCCGGTGGCGTTATTCGCCCTACGGCAATTGGG
GGGTAGGGCGAATAACCGCAACGCGGTTATCCGCCGTTGTCGCTGGGTTT

>3 PA3572
CGCCTGAGCGCGGATCAGGCCGTTCGCTGTTTCAGTTCGGGCAGCACATGGGGCGCGCGCAGGCGCGCCTCGAGG
CGGTCGACGAAACGCCGGGCCTCGCTTTCGCTACGGAAGTTGACGGCCTGCTGGTCGAGGCAGACTTGCCAGCAT
TGGCCTTTCGGGGTTTGCGACGGGCGGACGAGGATGTTCATGGCGATTCA

>4 PA3464
ATGTCGGGAAAGTCTAGCCGCCTGGCGCCTGGGCGCCATTGATGGCGGTCATGGGGCCGGCCCGGCGTGGATAGA
CATTGGTCCGATGGCGCTATCGGAGAGTTCCGATGTCCAGGCAGGACAGTGCCCTGGGTGTCCCGCATTGGTAGG
CTTGCCGGCACCGAGTCGAGACAAGAGCGAAAAGCCGATGAAGTTGCTGT

>5 PA3464
GAGCTGGGGCGCAGTCCGGCTTTTTCGGGGTGTGTAGGGCGAATAACGCCACGGGCGTTATCCGCCGATGTCGCG
GATGAGCGGCGGATGACCGCGAGCGGTTATTCGCCCTACGGAGCTGGGGCGCAGTCCGGCTTTTTCGGGGTGTGT
AGGGCGAATAACGCCATGGGCGTTATCCGCCGATGTCGCGGATGAGCGGC

>6 PA3397
TCCTTGCGCCCCGTCCGCGGGAGCGATGGGAGCCCTCGGCGGCCGGCGGCCGACCGATATCGTGTTATCCCATAT
CCATATTCTGGATAAGCATTATCCAGACAATTCATTTTGCGGATATTTTGGCAACCGATAAAATGCCGCGCAACT
GCGTCACGTCTGCTGCTGTTGGTGTGCGCCCGCGTTTTCCTAGGAGTCTT

>7 PA3391
ATCTCGCGGAACTCCGGCATGCCTTCGGCGTAGACGTCCGACAGGCGCTGGTAGTCGAGGTCGCGGAAGCTGATG
ATGTCGCCGAACTGGCGCAGTTGGACCCGATCGAAGATGCCACCGCGCACGTAGCCGGAACCCTTGAAGGAATAC
TCGCCGTCGCCGAGCACGACGAAGGCGTATTCGCCTGGCTTCAGCGACGC

>8 PA3342
AGCGTCTCTACGATCTCTACGAGCATGCCGATCGCGCGCTTTACGAAGCCAAGGCGTTCGGCCGTAACCAGGTGG
TGGCCTGAGTCCATGGCCGATTTTGTCCCTGTGGCTGGCGCCGCCGGTGACTGCGGGACGAGGCGGCGAATGCTA
GGTTCGACAGGAAAAAATGCCAGGCACAGCCGCTCTTCGCAGAGCTGCCG

>9 PA3341
CACTATTTCGGCACCTTGCTAGCCCAGACTTCCCGTGCTTGGCGCGCTGAACTCGACCGCCGCCTCAGTCACCTC
GGCCTGTCCCAGGCGCGCTGGTTGGTGTTGTTGCATCTGGCGCGCCATCGCGATTCGCCCACCCAGCGCGAGTTG
GCCCAATCGGTCGGGGTCGAAGGCCCGACCCTGGCGCGGCTGCTCGACGG

>10 PA3309
```

CGGCGATTTCCTCGCTGGCGCGCTCCTGGCAGACGCGATCGAAGGTGGCGCCTTCGACGTTGCTCGCCTCGACCT
TCTCGCGGGCCTTGGCCAGGACGTCATCGGCGGCTTTCAGCGCGGCTTCGCGCAGCGGCTCGGGGTTGTAGTACA
CCGCGTAGTCGGGAAGGTGGCGCAGCGGGCTGTCGACGATGGTGATGACG

>11 PA3083
TCGCTTCACTGTTTTCCATTGATGGAGTCCGCTTAATCGTGTTTCGCGAACCTCCCTGCGCAGGCTCTGCGAAGG
GACTTTTCGCGCCACCGGCGAAATTCGCCTGGCGCGTTTTGTCGGACACTTCCTGTTTTCTGGTCAGTGAATGAA
CAGTCATCCCGCAGGAAGTCCTGTTTTTTTCGCAATCCATTGAATTGCCT

>12 PA2742
ATCGCTCCCAAGAAGAAAAAGTAACCACCAGGGCACTGGCAGGCCTTGCGGTTATGCGTAATCACTCAATGCGGA
GTATCCGAACATGCCAAAGATGAAGACCAAAAGTGGCGCGGCCAAGCGCTTCAAGAAGACTGCTGGTGGCCTCAA
GCACAAGCACGCCTTCAAGAGCCACATCCTGACCAAGATGACCACCAAGC

>13 PA2601
GGATGACATGCGGGGATTCACTCCGGGAAGTTCTGGCGGTACGGTAGCAAACGCTTTTTTATTCCTGAAATGCAT
TTTGCATCTAAGCTAATATGCGAGAAATGCAATTGGTGCCGGCCCCATGAAAATCGATGACATCGACGCCTTCGT
CGCGGTGATCCGCAACGCTTCCCTGAGCCAGGCCGCCGAGAGCCTGGGCC

>14 PA2601
CGGCTACGCATAGTGTCCGCCTTGGGAAACGAAAAGACGGAAAAGCTAAATATGCGCTTTTAGCATATTAAGATA
TGACAAAAATGAATTTCCATCATGTAGTTTCAGCCTGTAGCTTCTAAGAAAAGCACCCATGCAGATGTTTTTCTT
ATAACGACAGCCGCCAACCTTATCAGTCGAGCGGCCCAGGCGAGGGCAAC

>15 PA2147
TGACCAGCACGCTCTCGCCGGCCGCGTTGACCAGGCGGAAGGTGTGCACGCCGAAGCCCTGCATGTTGCGGTAGG
CGATCGGAATGGCGCGATCGGACATCAGCCACATGACCATGTGCGCCGACTCCGGGGTCAGCGAAACGAAGTCCC
AGAAGGTGTCGTGGGCAGACGCTCCGGTGGGAATCTCGTTGTGCGGTTCG

>16 PA1414
CTGGCCAGCGCCGCAGGCGGAGGCCAGAGCGTCGGGGAACCGGCTCAGCGACGTTGCCGCGCGGCGAGGCGCCGG
CGTTGCTCGTCGTTTTCCACCGGGATGGGTTGCATGCGCGGGGGCTCGATCAGGCCAAGGGCGACACCGAGTTGC
CACGCCAGGTTTTGCAGCCAGAGTTTCATGACAGGCCCCTTGTCCAAGGG
underline

>17 PA1196
CAGGCCGCTGGCACGGTTGTAGTAGACGAAGCGACCGGAGGGGTCGACCACCGTGACCGGTTGCCGCAGGCCGTC
GAAAATCGGCGCCAGGCAGTCCACCAACCAGGCCAGTTCACCGCTTCGCGTGGAAATCGTCGGTTCCTCGACAAG
CGCCATGCTCTCCCCCTCGGTTTCCCGGATGTCGAAATCTCGTCAGTTGT

>18 PA0928
TGGGTCCAGCTGTCCGACATGCGCGCCCAGTTGATCGAGCGCGGGCAACTGATCGCCGAACAACTGGCGCCGCTG
GCCGCCACCGCGCTGGCGCGAAAGGATACCGCCGTGCTCAACCGCATCGCCAACGAGGCGCTGGACCAACCGGAC
GTGCGCGCGGTGACCTTCCTCGACGCCCGCCAGGAACGCCTCGCCCATGC

>19 PA4610
CTGGGCGTGCATGCGCGCCTGCGCCTGATCCCGAACCTGGACGCCGAGAGCCTGCCGAAGCTGGGCTGGCACATC
GCCGCGATCACCCTGGTGGGCCTGGGTTTCGTCGCGGTCGGACAGAGTTTCCGTTTCGGCGGCGCCTTCTGAGCA
TGCCCACATGAAAAAGGGCGCCGGAGGCGCCCTTTTTCATGCCGTTGTCG

>20 PA4772
GAAGCGGCGGATAGCGCCGCTGGCGCTGTTCGCCCTACCTCACGACTCCCACCG*GTAGGGCGAATA*ACCGCTCGC
GGTCATCCGCCGGGCCATGCTCCGCAGAAGCGGCGGATAGCGCCGCTGGCGC*TATTCGCCCTAC*GTCAGGCGAAA
TGGCTGCTCCGGCGTCGTCGGCCCGCGCCGGGTCGGGCAAAAAATCCGTC

>21 PA5208
GGCGCTGGCGTCCACCGAGCGCTGCACATAGGCGAGCATCTGGCCTTGTAGCGGCAACGGGATGCGCATGCGCCG
GCCCAGCATCAGCCCGGCGATGTCCTTGGCGCGATTGGCGACTTTGTCCTGTACACTGAGCAGCTCCAACAGATC
GGAGCGCGGCACCGGAAGGAACAGGCTCTTGGGCAGGTGCATCCGGACGT

>22 PA5243

GCGGCCTGCTTGGCGAAGTAGGTCAGAATGCCATCGGCGCCGGCACGTTTGAAGGCGGTAAGGGATTCGAGGATC
ACCGATTCGGCCAGCCAGCCGTTCTGGATGGCGCCCATGTGCATCGCATACTCGCCGCTGACCTGGTAGACAAAG
GTCGGGGCGCGGAATTCATCCTTCACCCGACGCACGATGTCGAGGTAGGG

>23 PA5294
GGCGGCAGCCTCGAAGGCGCGCAGGGCATACAGCGGGGGCAGGCGACGGGTCATTGGGGAATTACCTCTTTCATG
ACTTTTACTCATGCCTGGCATCTTGTTTTTCCTTTTGTGGAGCCATGCCAGTCGCACCGAGAATAGCCGCATTCT
CAACGCCGACAGGCCATCGCGGCAACCATCTCCTCCTGAGCCTTACTCAT

>24 PA3026
GACCAGCTTCTCGTAGGGCGTGATGCGGCTCATGGCCTCGCCCAGGGTGGCGCAGTTCATCGCGATGTAGCCGAG
CACGCTCCAGGAGCCGGGCTGGACGAAACGCGCCGAATGCAGGCCGAACAGGGGATCGCCGGAATGCTCGCAGAA
ATAGCCGAGCAGGTGCTCGTGGGCTTCGCCCGGCAGGCGCTGGCGATTGTCGTTGAGCTGGTCGGCCTGGAGGCC
GGCGGCGGCCAGGGCAGGGGCGATGTCCAGGCCGAGCCGTTCGGCATGGCGCAGGTATTTCAGCAGGGCCGGAAC
CGAGGTGGAGCCAAGGGTCTGCATGGTCGTTGTTCTTGTCGTGGCGGGTGGAACAGGCCGTCCGGCGTGCGTGCC
GGCTGTCTTGATCGGTCACAGGGAGTGTCTCGATCCGACAGTGACAGGCGAGGGCGGCTGGCTAGTATAGGCAGC
CAGAAAGGTTCCGATAAACAGGTTGCCGCGACAAGCGAGGGGAGAGGTTC
